# Tailoring rhodium-based metal-organic layers for parahydrogen-induced polarization: achieving 20% polarization of ^1^H in liquid phase

**DOI:** 10.1093/nsr/nwae406

**Published:** 2024-11-13

**Authors:** Jiawei Chen, Qi Zhang, Tao Chen, Zeyu Zheng, Yuhang Song, Huichong Liu, Ziqiao Chen, Jing Wang, Haoshang Wang, Huijun Sun, Xinchang Wang, Zhong Chen, Cheng Wang, Zhongqun Tian

**Affiliations:** State Key Laboratory of Physical Chemistry of Solid Surfaces, School of Electronic Science and Engineering, Department of Chemistry, College of Chemistry and Chemical Engineering, Xiamen University, Xiamen 361005, China; State Key Laboratory of Physical Chemistry of Solid Surfaces, School of Electronic Science and Engineering, Department of Chemistry, College of Chemistry and Chemical Engineering, Xiamen University, Xiamen 361005, China; State Key Laboratory of Physical Chemistry of Solid Surfaces, School of Electronic Science and Engineering, Department of Chemistry, College of Chemistry and Chemical Engineering, Xiamen University, Xiamen 361005, China; State Key Laboratory of Physical Chemistry of Solid Surfaces, School of Electronic Science and Engineering, Department of Chemistry, College of Chemistry and Chemical Engineering, Xiamen University, Xiamen 361005, China; State Key Laboratory of Physical Chemistry of Solid Surfaces, School of Electronic Science and Engineering, Department of Chemistry, College of Chemistry and Chemical Engineering, Xiamen University, Xiamen 361005, China; State Key Laboratory of Physical Chemistry of Solid Surfaces, School of Electronic Science and Engineering, Department of Chemistry, College of Chemistry and Chemical Engineering, Xiamen University, Xiamen 361005, China; State Key Laboratory of Physical Chemistry of Solid Surfaces, School of Electronic Science and Engineering, Department of Chemistry, College of Chemistry and Chemical Engineering, Xiamen University, Xiamen 361005, China; State Key Laboratory of Physical Chemistry of Solid Surfaces, School of Electronic Science and Engineering, Department of Chemistry, College of Chemistry and Chemical Engineering, Xiamen University, Xiamen 361005, China; College of Biology and Environmental Engineering, Zhejiang Shuren University, Hangzhou 310015, China; State Key Laboratory of Physical Chemistry of Solid Surfaces, School of Electronic Science and Engineering, Department of Chemistry, College of Chemistry and Chemical Engineering, Xiamen University, Xiamen 361005, China; State Key Laboratory of Physical Chemistry of Solid Surfaces, School of Electronic Science and Engineering, Department of Chemistry, College of Chemistry and Chemical Engineering, Xiamen University, Xiamen 361005, China; State Key Laboratory of Physical Chemistry of Solid Surfaces, School of Electronic Science and Engineering, Department of Chemistry, College of Chemistry and Chemical Engineering, Xiamen University, Xiamen 361005, China; Innovation Laboratory for Sciences and Technologies of Energy Materials of Fujian Province (IKKEM), Xiamen 361005, China; State Key Laboratory of Physical Chemistry of Solid Surfaces, School of Electronic Science and Engineering, Department of Chemistry, College of Chemistry and Chemical Engineering, Xiamen University, Xiamen 361005, China; Innovation Laboratory for Sciences and Technologies of Energy Materials of Fujian Province (IKKEM), Xiamen 361005, China; State Key Laboratory of Physical Chemistry of Solid Surfaces, School of Electronic Science and Engineering, Department of Chemistry, College of Chemistry and Chemical Engineering, Xiamen University, Xiamen 361005, China; Innovation Laboratory for Sciences and Technologies of Energy Materials of Fujian Province (IKKEM), Xiamen 361005, China; State Key Laboratory of Physical Chemistry of Solid Surfaces, School of Electronic Science and Engineering, Department of Chemistry, College of Chemistry and Chemical Engineering, Xiamen University, Xiamen 361005, China; Innovation Laboratory for Sciences and Technologies of Energy Materials of Fujian Province (IKKEM), Xiamen 361005, China

**Keywords:** metal-organic layers, heterogeneous catalysts, parahydrogen-induced polarization, hyperpolarization, nuclear magnetic resonance

## Abstract

Heterogeneous catalysts for parahydrogen-induced polarization (HET-PHIP) would be useful for producing highly sensitive contrasting agents for magnetic resonance imaging (MRI) in the liquid phase, as they can be removed by simple filtration. Although homogeneous hydrogenation catalysts are highly efficient for PHIP, their sensitivity decreases when anchored on porous supports due to slow substrate diffusion to the active sites and rapid depolarization within the channels. To address this challenge, we explored 2D metal-organic layers (MOLs) as supports for active Rh complexes with diverse phosphine ligands and tunable hydrogenation activities, taking advantage of the accessible active sites and chemical adaptability of the MOLs. By adjusting the electronic properties of phosphines, TPP-MOL-Rh-dppb (TPP = tris(4-carboxylphenyl)phosphine), featuring a *κ*_2_-connected di(phosphine) ligand, generated hyperpolarized styrene achieving an over-2400-fold signal enhancement and a polarization level of 20% for ^1^H in methanol-*d*_4_ solution. The TPP-MOL-Rh-dppb effectively inherited the high efficiency and pairwise addition of its homogenous catalyst while maintaining the heterogeneity of MOLs. This work demonstrates the potential of 2D phosphine-functionalized MOLs as heterogeneous solid support for HET-PHIP.

## Teaser

Metal-organic layers, functioning as heterogeneous 2D supports for rhodium complexes with diverse phosphine ligands, enable tunable hydrogenation activities and achieve 20% polarization in the liquid phase under mild conditions.

## INTRODUCTION

Nuclear magnetic resonance (NMR) provides high-resolution structural insights into dynamic molecules and finds extensive usage in chemistry, biology and medical imaging [1–3]. One major limitation, however, is its low sensitivity due to the small thermal polarization of nuclear spins, restricting NMR's broader application scope [4]. To combat this, two primary sensitivity enhancement strategies have been proposed. The first involves increasing polarization at thermal equilibrium by using a high-strength magnetic field, but the enhancement is limited and the cost is high [5]. The second and potentially more effective approach is hyperpolarization, which alters the Boltzmann distribution of nuclei and boosts nuclear magnetic sensitivity by several orders of magnitude [6,7]. Popular hyperpolarization techniques include dynamic nuclear polarization [8,9], spin exchange optical pumping [10], hyperpolarization through nitrogen-vacancy color centers and parahydrogen-based hyperpolarization [11,12].

Parahydrogen-induced polarization (PHIP) and signal amplification by reversible exchange (SABRE) have garnered significant attention due to their high enhancement factors and cost-effectiveness [13–16]. Parahydrogen (*p*-H_2_), a nuclear spin isomer of molecular hydrogen, possesses a high degree of nuclear spin polarization. In SABRE, *p*-H_2_ and the substrate are transiently bound to an organometallic complex, allowing spontaneous transfer of the spin order from *p*-H_2_ to the substrate. The enhancement of NMR signal through PHIP involves hydrogenating an unsaturated molecule through pairwise addition of *p*-H_2_ [13,17]. This method shows promise for sensitive detection of liquid-phase reaction intermediates [18,19], metabolic processes [20] and producing liquid-phase contrasting agents for magnetic resonance imaging (MRI) [21,22].

For PHIP, an efficient catalyst is critical for achieving a high polarization level. The catalyst must add *p*-H_2_ to the substrate in a pairwise manner, which means both hydrogen atoms in the product must come from the same *p*-H_2_ molecule [23,24]. Homogeneous catalysts [25,26], like Rh(PPh_3_)_3_Cl (Wilkinson's catalyst) or Rh(COD) (dppb)BF_4_ (dppb = 1,4-bis(diphenylphosphino)butane; COD = 1,5-cyclooctadiene), show high efficiency of hydrogenation and ensure pairwise addition [17,19]. Several works have reported methods to remove homogeneous catalysts to create hyperpolarized MRI contrasting agents [21–29], but these procedures are rather complicated, increasing the risk of losing polarization during processing. Heterogeneous catalysts, on the other hand, can be removed by simple filtration [21]. Thus, developing an efficient heterogeneous PHIP (HET-PHIP) catalyst is of great interest for the application of PHIP in MRI [3,30,31]. Metal nanoparticle-based HET-PHIP (MNP-HET-PHIP) catalysts have achieved high signal enhancement [12], but their surfaces contain various types of active sites and the pairwise selectivity is still limited (<20%). Moreover, high enhancement factors using HET-PHIP catalysts were achieved mostly using gas-phase unsaturated molecules such as propene under relatively high temperatures (from 80 to 500°C) [32–34]. Signal enhancements in solution using HET-PHIP catalysts are still low [35–37].

To combine the advantages of higher pairwise selectivity of homogeneous catalysts and the easy removal of heterogeneous catalysts, an alternative approach is to immobilize homogeneous catalysts with superior catalytic activity on solid supports to create heterogeneous active sites [38,39]. Various porous solids have been explored as supports in HET-PHIP, including phosphine ligand-functionalized silica gel [34,40–42] or polymer [40], and metal-organic frameworks (MOFs) [43] (Fig. S1a and b). However, for porous solids, substrate diffusion rate in the porous channels is limited [43,44], which could hinder the reaction of a LM(H)_2_ intermediate with the substrate [43]. Furthermore, molecules inside porous materials have lower T1 relaxation times compared to their bulk fluids [45,46]. This is due to the interaction between the porous walls and the restricted motion of the molecules, accelerating the relaxation processes and resulting in faster relaxation of hyperpolarized signals. Designing a novel catalyst with high PHIP efficiency and quick release of hyperpolarized product is both significant and challenging.

Metal-organic layers (MOLs), 2D analogs of MOFs [47–49], offer a solution (Fig. S1c). As a monolayer, MOLs provide more accessible active sites, overcoming the diffusion limitations of MOFs [44,50–52]. This study introduces Rh complexes with varying numbers of phosphine ligands with diverse electronic structures anchored onto a phosphine-based MOL (TPP-MOL) [53,54]. By adding additional phosphine ligands, we modulated the activity and depolarization lifetime of the Rh sites, maintaining the spin order of *p*-H_2_ in the HET-PHIP. The phosphine-functionalized MOL opens new avenues for the screening of highly active heterogeneous PHIP catalysts.

## RESULTS AND DISCUSSION

### Catalyst synthesis and characterization

**Figure 1.**
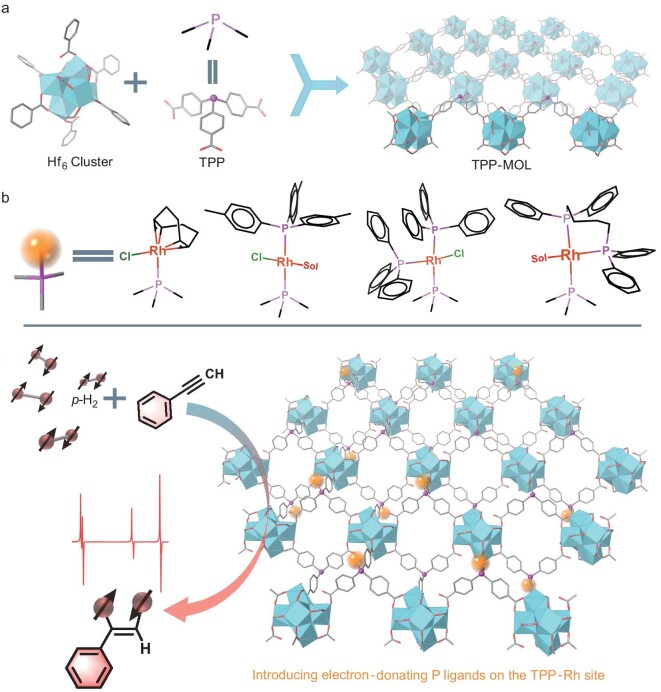
(a) Structure of the TPP-MOL. (b) Schematic representation of Rh complexes within MOLs, showing variations in the number and electronic structures of phosphine ligands for PHIP application.

**Figure 2.**
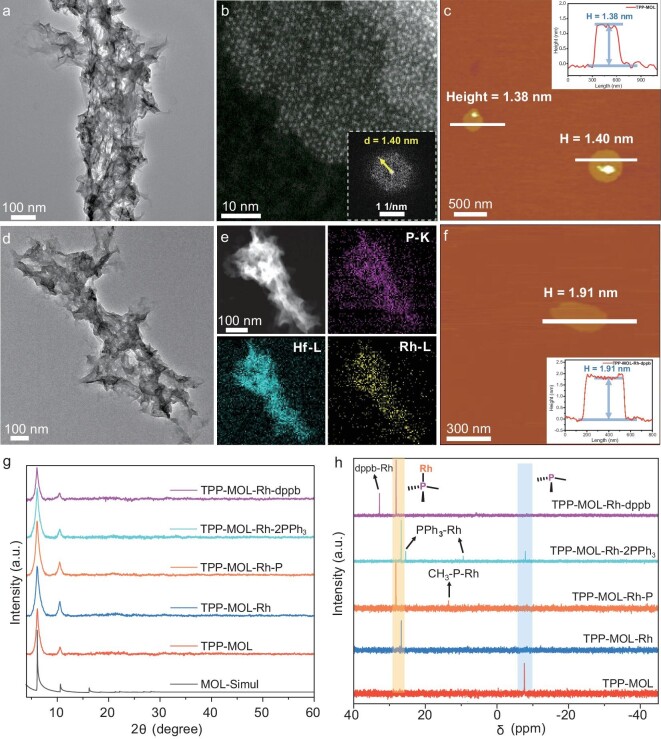
(a) TEM, (b) HAADF and (c) AFM images of the TPP-MOL. The inset in (b) is the fast Fourier transform (FFT) pattern. (d) TEM, (e) HAADF and EDX mapping, and (f) AFM images of TPP-MOL-Rh-dppb. The insets in (c) and (f) depict the height profile along the white line (H represents height). (g) PXRD patterns of various TPP-MOL derivatives alongside the simulated pattern of the monolayer model. (h) ^31^P-NMR spectra of the digested MOL catalysts.

The 2D phosphine-functionalized TPP-MOL was synthesized via a one-pot solvothermal reaction between tris(4-carboxylphenyl) phosphine (TPP) and HfCl_4_ under N_2_ protection at 120°C for 48 h [54]. In the MOL structure, the TPP ligands connected with Hf_6_-oxo secondary building units (SBUs) via carboxylate linkages, forming a 3,6-connected 2D network of *kgd* topology [54] (Fig. 1). The TPP-MOL showed ultrathin wrinkled films as observed by transmission electron microscopy (TEM) (Fig. 2a). High-angle annular dark field (HAADF) imaging revealed a hexagonal arrangement of Hf_6_ SBUs, with a (100) inter-plane distance of 1.40 nm (perpendicular to the monolayer norm), corresponding to inter-SBU spacing of 1.66 nm (Fig. 2b). Atomic force microscopy (AFM) topography confirmed the monolayer with a thickness of ∼1.40 nm (Fig. 2c). ^31^P-NMR analysis (Figs S2, S3) of the TPP-MOL digested by tetra-*n*-butylammonium fluoride (TBAF) aqueous solution showed a peak at δ -7.65 ppm, indicating that most of the trivalent phosphine ligand was not oxidized under the N_2_ protection [55]. The molar ratio of Hf/ligand was determined to be 3.4/1 by thermogravimetric analysis (TGA) (Fig. S4), close to the expected value of 3/1 in the model structure.

To empower MOLs with the ability to catalyze the hydrogenation reaction, TPP-MOL was modified with a variety of Rh complexes, including [Rh(COD)Cl]_2_, Rh(COD)(CH_3_-P)Cl (CH_3_-P = tris(4-methylphenyl)phosphane), Rh(PPh_3_)_3_Cl and Rh(COD)(dppb)BF_4_. These complexes, featuring phosphine ligands with varying electron-donating properties, led to preparation of mono(phosphine)-Rh (TPP-MOL-Rh), di(phosphine)-Rh (TPP-MOL-Rh-P) and tri(phosphine)-Rh (TPP-MOL-2PPh_3_ and TPP-MOL-Rh-dppb) variants, each anchored on the MOL solids (Fig. S1c and Fig. 1b). The incorporation of these diverse phosphine ligands at the TPP-Rh sites effectively tuned their electronic structures [56,57]. Inductively coupled plasma-optical emission spectroscopy (ICP-OES) analysis confirmed nearly full loading of Rh centers on the TPP sites in the TPP-MOL-Rh catalyst (99.9% with respect to TPP, 6.5 wt% Rh). In contrast, the introduction of multi(phosphine) ligands resulted in competitive coordination [40] between the immobilized phosphine and the phosphine in solution, leading to reduced Rh loadings of 73.5% (4.5 wt% Rh), 68.2% (4.2 wt% Rh) and 39.8% (2.5 wt% Rh) relative to TPP, respectively (Table S1). Energy-dispersive X-ray (EDX) mapping revealed uniform distribution of Rh, Hf and P elements across the ultrathin wrinkled films of the four catalysts (Fig. 2d and e and Figs S5–S7). AFM imaging of TPP-MOL-Rh-dppb showed that the ultrathin morphology was preserved, with a thickness of ∼1.91 nm after post-synthetic modification (Fig. 2f), which was attributed to the post-modified Rh-dppb site expanding the thickness of the monolayer. Powder X-ray diffraction (PXRD) data indicated that these four catalysts maintained the topological structure of TPP-MOL [58] (Fig. 2g).

To investigate the coordination environment of multi(phosphine) ligands at the Rh site, NMR analysis was performed on the MOL after digestion with either TBAF or a saturated potassium phosphate (K_3_PO_4_) solution. The chemical shift of ^31^P from TPP-MOL-Rh (δ = 26.73 ppm) exhibited a downfield shift [59] compared with uncoordinated TPP at δ -7.65 ppm, which disappeared in TPP-MOL-Rh (Fig. 2h and Fig. S8).

To modulate the electronic properties and hence the hydronation reactivities of the MOLs, in addition to TPP, the TPP-MOL-Rh was modified by the electron-rich tris(4-methylphenyl)phosphine (CH_3_-P) ligand on the Rh site as TPP-MOL-Rh-P, as evidenced by the appearance of new peaks corresponding to the CH_3_-P-Rh species in the ^1^H-NMR (δ = 7.42, 7.33 and 2.33 ppm) and ^31^P-NMR (δ = 13.54 ppm) spectra after K_3_PO_4_ digestion (Fig. 2h and Fig. S9). Moreover, the TPP-MOL-Rh-2PPh_3_ catalyst was obtained by directly treating TPP-MOL with Rh(PPh_3_)_3_Cl. The ^31^P-NMR spectrum of the digested MOL exhibited the emergence of two peaks at δ 25.48 and 9.42 ppm, corresponding to the coordination of two PPh_3_ ligands at the TPP-Rh sites (Fig. 2h).

Previous reports showed that incorporating a *κ*_2_-connected di(phosphine) ligand into a cationic Rh complex leads to an elevated polarization level in hydrogenated products via PHIP [60,61]. This enhancement may be attributed to the accelerated conversion of the dihydride intermediate together with a reduced rate of depolarization [61,62]. We thus further synthesized TPP-MOL-Rh-dppb catalyst by reacting Rh(COD) (dppb)BF_4_ with TPP-MOL in a 3 : 1 (Rh : TPP) stoichiometric ratio. A new peak appeared at δ 32.81 ppm in the ^31^P-NMR spectrum of the digested MOLs (Fig. 2h), corresponding to the chelating dppb ligands at the TPP-Rh site. Additionally, a more detailed analysis of the phosphine ligands was performed with the ^1^H-NMR spectrum (Figs S10 and S11). In X-ray photoelectron spectroscopy (XPS) analysis, the P 2p peak shifted to higher binding energy after modification with a series of Rh complexes (from 130.8 eV to 132.5 eV). The Rh 3d peaks were slightly widened for TPP-MOL-Rh-2PPh_3_ and TPP-MOL-Rh-dppb with multi(phosphine)-Rh sites (Fig. S12).

**Figure 3.**
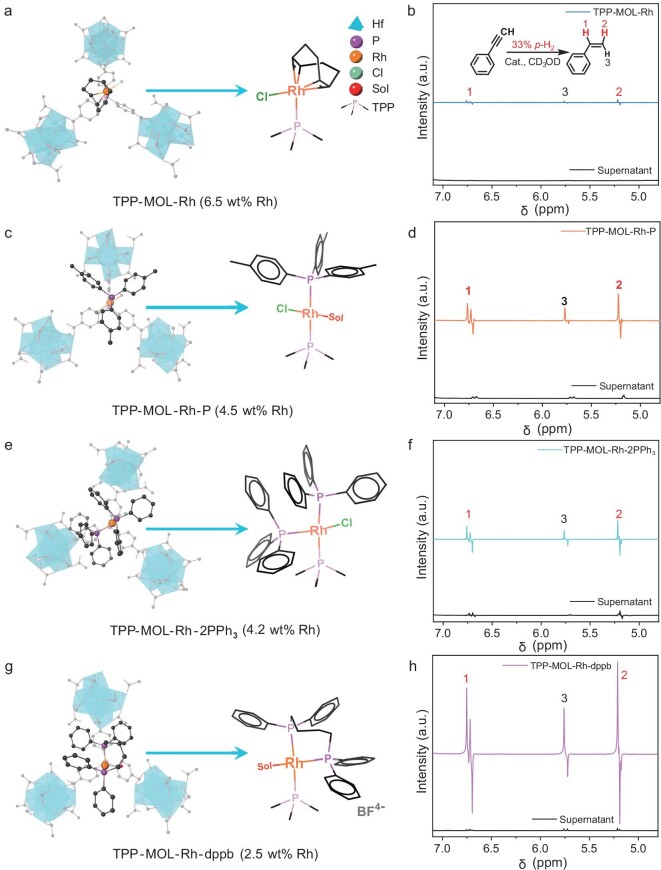
Structures and PASADENA ^1^H-NMR spectra of (a, b) TPP-MOL-Rh, (c, d) TPP-MOL-Rh-P, (e, f) TPP-MOL-2PPh_3_ and (g, h) TPP-MOL-Rh-dppb catalysts with Rh loading of 0.44 mol%. The spectra of the separated supernatant after the reaction are included (below), normalized to signals in the aromatic region and presented on the same vertical scale.

By controlling the number of phosphine ligands with different electron-donating properties, we constructed structurally diverse Rh sites (TPP-MOL-Rh-P_m_). The molecular structures of local multi(phosphine)-Rh species are comprehensively illustrated in Fig. 3a, c, e and g, where the coordination of Cl^−^ and solvent was determined by the precursor type of Rh and the geometric configuration of Rh (Ⅰ).

### PHIP activity of MOL-based catalysts using 33% *p*-H_2_

The hyperpolarization of phenylacetylene was conducted inside a 500 MHz Varian liquid NMR spectrometer (Agilent Technologies, Santa Clara, CA, USA) in CD_3_OD, employing the PASADENA method (parahydrogen and synthesis allow dramatically enhanced nuclear alignment [7,30,63]). Parahydrogen was enriched by passing normal H_2_ through a copper tube containing FeO(OH) catalyst cooled by two different methods. For *p*-H_2_ enriched using liquid N_2_ (77 K), its ratio is determined to be 33% by ^1^H NMR spectra, and for *p*-H_2_ enriched using compressor (35 K), its ratio is 96% (for details, please see section 11 and Fig. S13 in the online supplementary data). The ratios of *p*-H_2_ used in each experiment are specified in the corresponding context.

**Figure 4.**
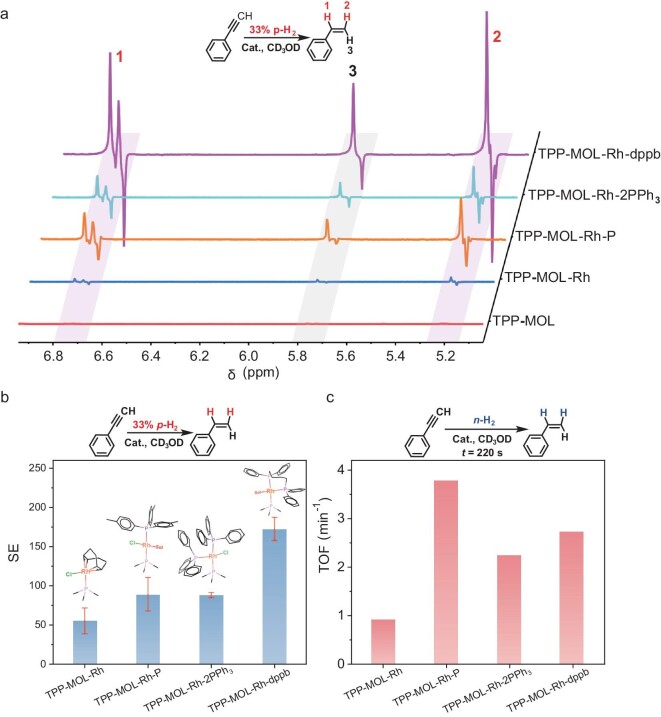
(a) Summary of normalized PASADENA ^1^H-NMR spectra using 33% *p*-H_2_. (b) Signal enhancement (SE) and (c) turnover frequency (TOF) of TPP-MOL-Rh, TPP-MOL-Rh-P, TPP-MOL-2PPh_3_ and TPP-MOL-Rh-dppb catalysts in phenylacetylene hydrogenation. The SE error bars in (b) were derived from multiple PASADENA experiments in Table S2. TOF was calculated based on the ^1^H-NMR data of normal H_2_ (*n*-H_2_) hydrogenation.

The reaction mixture containing the TPP-MOL catalyst (Rh loading of 0.44 mol% with respect to the substrates) was equilibrated at 50°C. Subsequently, *p*-H_2_ (33%) gas was bubbled into the NMR tube until it reached a constant pressure of 3 bar, and the hyperpolarized ^1^H-NMR spectra were acquired immediately. Antiphase doublets, characteristic of PHIP in PASADENA were observed in all the hyperpolarized ^1^H-NMR spectra catalyzed by the MOL catalysts at δ 6.72 ppm and 5.20 ppm (Fig. 3b, d, f and h and Fig. 4a). The signal enhancement (SE) factor is calculated using the gas-liquid-solid phase formulae proposed by Igor V. Koptyug and co-workers (section 12 in the online supplementary data) [36]. Different from gas-solid phase hydrogenation, the hydrogenated substrate remains in solution after the hydrogenation reaction, which leads to a large signal in thermal equilibrium, so we used the Igor V. Koptyug method and measured the increase in the thermal equilibrium signal corresponding to the bubbling time as the denominator of the SE factor calculation.

The hydrogenation activity of a catalyst is positively correlated to the electron-donating properties of the phosphine ligands [62,64,65]. Experimentally, introducing an electron-donating CH_3_-P ligand in TPP-MOL-Rh-P (Turnover frequency (TOF) = 3.8 min^−1^) enhanced the activity nearly 4-fold compared with TPP-MOL-Rh (TOF = 0.9 min^−1^), along with a higher SE (89 ± 21 for TPP-MOL-Rh-P vs. 55 ± 17 for TPP-MOL-Rh) (Fig. 3b and d and Fig. 4, Table S2, Entry 1, 2).

For Rh sites coordinated with three phosphorus atoms, TPP-MOL-Rh-dppb (TOF = 2.7 min^−1^) showed comparable hydrogenation activity to TPP-MOL-2PPh_3_ (TOF = 2.2 min^−1^) but significantly higher SE (173 ± 15 vs. 88 ± 3) (Fig. 3f and h, Fig. 4, Fig. S14, Table S2, Entry 3, 4). Due to the low yield of styrene at several minutes, no detectable ethylbenzene from deep hydrogenation was observed. The specific reactivities are summarized in Table S2.

Based on previous reports [60,61], we reasoned that the hydrogenation process using TPP-MOL-Rh-dppb catalyst with the *κ*_2_-connected di(phosphine) ligand involves initial substrate adsorption followed by oxidative addition of H_2_ to the Rh center (unsaturated route, see in Fig. S15a) and then rapid pairwise H-addition to the substrate. In contrast, TPP-MOL-Rh-2PPh_3_, akin to Wilkinson's catalyst, likely initially undergoes reversible oxidative addition of *p*-H_2_ to the Rh center before substrate adsorption, making it more prone to depolarization on the Rh center before addition to the substrate (Fig. S15b) [60,61,66].

**Figure 5.**
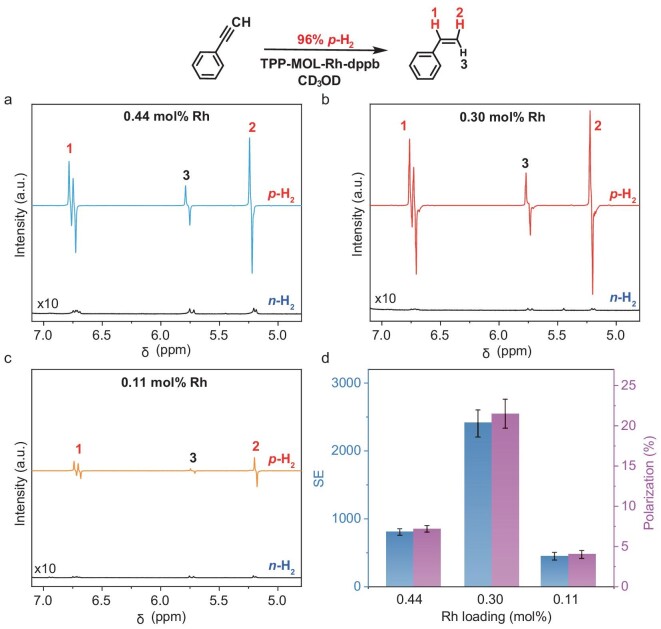
PASADENA ^1^H-NMR spectra using 96% *p*-H_2_ of TPP-MOL-Rh-dppb with Rh loading of (a) 0.44 mol%, (b) 0.30 mol% and (c) 0.11 mol%, respectively, normalized to signals in the aromatic region and presented on the same vertical scale. The spectra using *n*-H_2_ are included (below, vertically scaled by 10-fold). (d) Comparison of SE factor and proton polarization.

Among the four catalysts, TPP-MOL-Rh-dppb exhibited the highest SE in PASADENA experiments (Fig. 5). The SE factor of the hyperpolarized product using TPP-MOL-Rh-dppb catalyst with 33% *p*-H_2_ was slightly lower than that of the homogeneous counterpart (Rh(COD)(dppb)BF_4_) (SE = 173 ± 15 vs. SE = 204), and its yield is also lower than that of homogeneous catalyst within a reaction time of 220 s (yield = 4.4% vs. yield = 57.6%) (Fig. S16 and Table S2, Entry 4, 6). However, the PHIP activity of the TPP-MOL-Rh-dppb surpassed a MOF-based catalyst for gas-phase products (SE = 16) [43] and surpassed most of the previous heterogeneous catalysts for gas-solid-liquid phase or gas-solid phase products [36,67–69] (Tables S3, S4). We attributed the low enhancement of the porous heterogeneous catalysts to the accelerated relaxation of the fluids within the pores [49]. The above results highlighted the advantages of 2D MOLs without porous channels as support in PHIP applications. The MOL's accessible active sites facilitate the diffusion of hydrogenation products from the metallic catalytic centers to the solution, resulting in the preservation of hyperpolarization signals.

To demonstrate the experimental factors that could influence hyperpolarization, the effects of temperature and Rh loading on polarization activity were assessed. The relative intensity of the polarization signals at 50°C was obviously stronger than that at 25°C, indicating that higher temperature enhanced the overall rate of reaction (Fig. S17). Moreover, the effect of Rh loading was investigated by varying the amount of MOL catalysts (0.05–0.88 mol% Rh). The TPP-MOL-Rh-dppb catalyst exhibited the highest polarization signals at 0.44 mol% (Fig. S18). We postulated two reasons for the relatively weak polarization signal of products with higher Rh loading. First, higher Rh loadings led to the polymerization of phenylacetylene [36,70], as indicated by a yellow color after the reaction and the observation of polymerization products by a broad peak at δ 5.79 ppm (−C = CH−) [71] in the ^1^H-NMR spectrum (Fig. S19). Second, a higher Rh loading increased the probability of re-adsorption of the generated olefine product on the metal center, which may lead to faster depolarization and thus a reduction in the intensity of the polarization signal.

To expand the range of MOL-based catalysts for HET-PHIP, we introduced the more electron-rich 1,4-bis(dicyclohexylphosphanyl)butane (dhpb) ligand to obtain TPP-MOL-Rh-dhpb (22.5% with respect to TPP, 2.5 wt% Rh) (Fig. S20a–d). However, the dhpb ligand enhanced both the hydrogenation [62] and polymerization of phenylacetylene, as evidenced by the rapid transformation of the reaction solution to a deep orange color. Nevertheless, this catalyst with a relatively lower Rh loading (0.33 mol%) achieved a high SE of 202 using 33% *p*-H_2_, comparable to TPP-MOL-Rh-dppb (Fig. S20e, Table S2, Entry 5).

### Optimizing PHIP activity using 96% *p*-H_2_

Based on the aforementioned study, we further optimized the PASADENA experiments using 96% *p*-H_2_. The SE factor for styrene increased to 805 ± 49 using TPP-MOL-Rh-dppb with a Rh loading of 0.44 mol%, and ^1^H polarization level of 6.3 ± 0.4% (Fig. 5a and d and Table S5, Entry 1). The generated hyperpolarization signals of NMR were stronger in intensity than the thermal signals of the styrene (Fig. S21).

Subsequently, we reduced the usage of the MOL-based catalyst. Within the investigated range of 0.22–0.44 mol% Rh, a moderate loading of 0.30 mol% was chosen, which exhibited an increase of SE to 2404 ± 200 and a higher proton polarization level of 18.9 ± 1.5% (Fig. 5b and d, Fig. S22 and Table S5, Entry 2). In comparison, the polarization of ^1^H in SABRE is generally <15% (Tables S6 and S7), with one exception reported by the Simon Group recently using a heterogeneous SABRE catalyst [14,72,73]. However, the yield of styrene at a lower Rh loading of 0.30 mol% was reduced by over 2-fold compared to that of the Rh loading of 0.44 mol%. Moreover, a lower Rh loading of 0.11 mol% exhibited lower polarization (Fig. 5c and d, Figs S23, S24 and Table S5, Entry 3).

### Heterogeneity test

To assess the heterogeneity of the catalysts, we conducted the following tests:

First, after hydrogenation reactions with each MOL catalyst, the supernatant was separated and reacted with fresh phenylacetylene using 33% *p*-H_2_. No significant hyperpolarized signals were observed in most cases (Fig. 3b, d, f and h, Fig. S25), indicating minimal leaching of active Rh species. Only the TPP-MOL-Rh-2PPh_3_ catalyst showed low-intensity hyperpolarized signals, suggesting possible trace Rh leaching.

Second, ^31^P-NMR analysis of the separated supernatant after the reaction revealed no detectable leaching of any phosphine ligands (Fig. S26), confirming the stability of the MOL under the mild reaction condition.

Third, PXRD patterns and TEM images of the four catalysts after hydrogenation matched those of the initial MOL catalysts (Figs S27 and S28), indicating the preservation of the monolayer structure under catalytic conditions.

Fourth, the hyperpolarized signals obtained using the TPP-MOL-Rh-dppb catalyst maintained their relative intensity even after three cycles of reuse (Fig. S29). The decrease in hyperpolarization signal intensity observed after three cycles may be due to the loss of catalyst content, the reduction of catalyst active sites and a small amount of catalyst deactivation.

Fifth, ICP-OES analysis showed minimal Rh leaching (0.1%) from the TPP-MOL-Rh-dppb catalyst after the first reaction (Table S1). XPS data confirmed that the Rh oxidation state remained unchanged before and after the reaction (Fig. S30).

These results collectively demonstrated the heterogeneity of the MOL catalyst in the PHIP process.

### Scope of unsaturated substrates

We expanded the scope of unsaturated substrates using a TPP-MOL-Rh-dppb catalyst under a 33% *p*-H_2_ atmosphere. For 1-phenylpropyne, a polymerization-unfavored substrate, we observed improved hydrogenation efficiency, albeit with a relatively low SE (52) (Fig. S31, Table S8, Entry 2). This could be attributed to *J*-coupling between the parahydrogen protons and adjacent methyl groups [20], a phenomenon also observed in Rh/TiO_2_ systems [36].

In contrast, propargyl acetate, a hydrolyzable ester molecule relevant for MRI contrast agents [20,74], exhibited a high SE (183), despite lower hydrogenation activity (Fig. S32, Table S8, Entry 3). For styrene, the catalyst showed lower hydrogenation activity [75] with an SE of 107 (Fig. S33, Table S8, Entry 4).

### Pairwise hydrogenation mechanism

The PASADENA ^1^H-NMR spectra of hyperpolarized styrene from phenylacetylene hydrogenation using a MOL catalyst give strong signal enhancements for proton #1 and #2, indicating the preservation of spin order from the *p*-H_2_ protons in the hydrogenation products (Fig. 4a), consistent with the pairwise hydrogenation pathway observed in homogeneous systems [17,19,64].

**Figure 6.**
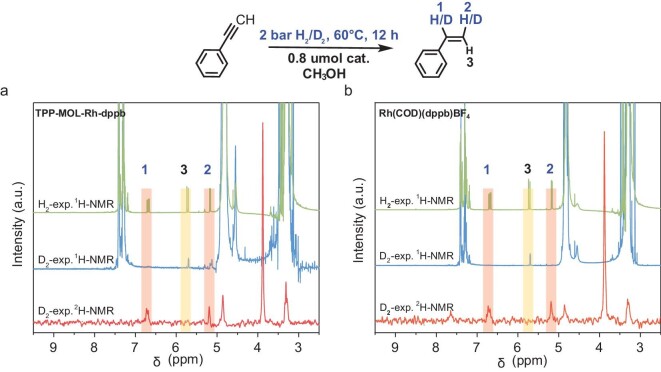
The normal H_2_ and D_2_ hydrogenation of (a) TPP-MOL-Rh-dppb catalyst and (b) Rh(COD) (dppb)BF_4_ catalyst (to account for the drift of deuterium chemical shifts, O_2_NPhCOOCD_3_ with its known chemical shift at ∼4 ppm was added for calibration).

To investigate the mechanism of hydrogenation of our catalysts, we conducted hydrogenation experiments using D_2_ (Fig. 6). The distribution of deuterium in the products showed that proton #1 and #2 of the vinyl group originated from the *syn*-hydrogenation of D_2_, ruling out *anti*-addition or isomerization as contributors to the polarized proton signal #3. Previous literature [76,77] suggests that this signal arises from a combination of dynamic scalar coupling (zero-quantum transitions), dynamic dipolar coupling (double-quantum transition) and a time-dependent scalar coupling process resulting from single bond rotation between the phenyl and double bond in styrene (Fig. S34, details are provided in the online supplementary data).

The combined PASADENA and D_2_ hydrogenation experiments provide compelling experimental evidence supporting the pairwise *syn*-hydrogenation mechanism in heterogeneous catalysts (Fig. S15a). These results are crucial for understanding the hydrogenation mechanism and guiding catalyst design for HET-PHIP.

## CONCLUSIONS

In this study, we demonstrated the use of MOL-based catalysts for HET-PHIP, producing hydrogenated molecules with significantly enhanced NMR signals. By anchoring Rh with various auxiliary ligands to the freely accessible phosphine sites on the TPP-MOL, we achieved controllable electronic structures of the Rh center conducive for HET-PHIP. The TPP-MOL-Rh-dppb catalyst, featuring a *κ*_2_-connected di(phosphine) ligand, was proved to be the most effective catalyst for inducing pairwise addition of *p*-H_2_ after the coordination of the unsaturated substrates, leading to remarkable signal enhancement in the hydrogenation of phenylacetylene under mild conditions. Further experiments are underway to address the current shortcomings of the catalyst, such as enhancing its hydrogenation conversion. The hyperpolarization of gas-phase substrates, such as propene, is also underway. This work not only highlights the potential of phosphine-based MOLs in developing highly active HET-PHIP catalysts, but also provides valuable insights into the pairwise hydrogenation mechanism. Furthermore, the principles of ligand environment tuning at anchored sites, as demonstrated here, have the potential to be applied to a broader range of challenging reactions.

## Supplementary Material

nwae406_Supplemental_File
