# Supplementary material for: Tailoring rhodium-based metal-organic layers for parahydrogen-induced polarization: achieving 20% polarization of 1H in liquid phase
Source: Natl Sci Rev. 2024 Nov 13;12(1):nwae406. doi: 10.1093/nsr/nwae406 (PMC11702662; doi:10.1093/nsr/nwae406)
Supplement: nwae406_Supplemental_File [file nwae406_supplemental_file.pdf]

Supplementary Information

Tailoring Rhodium-center on Metal-Organic Layers for Parahydrogen-Induced Polarization: Achieving 20% Polarization of ^{1}H in Liquid Phase

Content

1. Materials and methods 2
2. Synthesis of TPP ligand 3
3. Synthesis of MOL catalysts 4
4. PXRD simulation of TPP-MOL 6
5. Strategies for HET-PHIP 7
6. Analysis of composition for TPP-MOL 8
7. Rh loading amount of MOL catalysts 9
8. Morphology and elemental mapping of MOL catalysts 10
9. ^{1}H-NMR determination of multi(phosphine)-Rh sites 11
10. XPS analysis of MOL catalysts 13
11. Enrichment of parahydrogen and PASADENA ^{1}H-NMR experiments 14
12. Calculation of signal enhancement and yield 16
13. PHIP activity of MOL heterogeneous catalysts using 33% p-H_2 17
14. Structure and HET-PHIP activity of TPP-MOL-Rh-dhpb 26
15. PHIP activity of TPP-MOL-Rh-dppb catalyst using 96% p-H_2 27
16. Heterogeneity test of MOL catalysts using 33% p-H_2 33
17. Scope of unsaturated substrates using TPP-MOL-Rh-dppb 36
18. Pairwise hydrogenation mechanism 38
19. Simulated PHIP experiment of styrene 39
20. References 42

1. Materials and methods

Unless otherwise stated, all reagents were commercially available and used without further purification. Toluene was distilled prior to use. N, N-Dimethylformamide (DMF), H_2O, formic acid (HCO_2H) was degassed by bubbling N_2 to remove dissolved oxygen. CD_3OD for parahydrogen-induced polarization (PHIP) test underwent degassing through freeze-pump-thaw method. Resistance vials equipped with PTFE-lined caps were obtained from WHEATON (USA).

Transmission electron microscopy (TEM) images, high-angle annular dark field (HAADF) images and energy-dispersive X-ray (EDX) mapping were taken using Tecnai F20 and Tecnai F30 scanning transmission electron microscopy (STEM, Philips-FEI) operating at an electron acceleration voltage of 200 kV and 300 kV. Atomic force microscopy (AFM) images were acquired using a Cypher S Asylum Research atomic force microscopy in tapping mode. Powder X-ray diffraction (PXRD) patterns were obtained using a Japan Rigaku IV X-ray diffractometer equipped with Cu Kα radiation (λ = 1.54178 Å). ^{1}H-NMR and ^{31}P-NMR spectra were recorded using a Bruker 400 DRX spectrometer at 400 MHz and a Bruker 500 DRX spectrometer at 500 MHz. ^{2}H-NMR spectra were recorded on a Bruker 400 DRX spectrometer at 400 MHz. Thermogravimetric analysis (TGA) was carried out in air using a Shimadzu TGA-50 equipped with an alumina pan. Inductively coupled plasma-optical emission spectroscopy (ICP-OES) analyses of sample digested with concentrated hydrochloric acid and nitric acid (3/1, v/v) were acquired using a Thermo Fisher iCap 7000 instrument. X-ray photoelectron spectroscopy (XPS) tests were carried out using a Thermo Fisher spectrometer, with the binding energy corrected by the C 1s peak (284.8 eV). PHIP tests were performed using a 500 MHz Varian liquid NMR spectrometer (Agilent Technologies, Santa Clara, CA, USA) equipped with a gas flow meter and a heating module.

2. Synthesis of TPP ligand

Tris(4-carboxylphenyl)phosphine oxide (TPO) was synthesized following the method reported previously.[1] TPO (2 g, 4.9 mmol) and anhydrous toluene (34 mL) were added to a 250 mL Schlenk tube. The reducing agent $HSiCl_3$ (22 mL, 218 mmol) was then added to the suspension. The mixture was heated at 130 ℃ for 3 days under a nitrogen atmosphere. Upon cooling, the solvent was evaporated and the resulting yellow residue was treated with concentrated NH_3 solution. The aqueous phase containing the product was separated by centrifugation. The filtrate was then acidified with dilute HCl to obtain a white solid. The collected solid was washed with deionized water and dried at 60 ℃ under vacuum to obtain the TPP ligand (1.18 g, 62% yield).

^{1}H NMR (500 MHz, DMSO-d_6) δ 13.13 (s, 3H), 7.97 (dd, J = 8.3, 1.6 Hz, 6H), 7.39 (t, J = 7.8 Hz, 6H).

^{31}P NMR (202 MHz, DMSO-d_6) δ -6.37.

3. Synthesis of MOL catalysts

Synthesis of TPP-MOL

The synthesis of TPP-MOL followed the methods described in previous work.[2] $HfCl_4$ (12 mg, 0.038 mmol) and H_3TPP (11.7 mg, 0.03 mmol) were dissolved in a solution of DMF (0.833 mL), HCO_2H (0.12 mL) and H_2O (0.025 mL) in a pressure-resistant vial. The mixture was heated at 120 ℃ under N_2 atmosphere for 2 days. Upon cooling, white precipitates of TPP-MOL $Hf_6O_8H_4(TPP)_2(HCO_2)_3(H_2O)_2(H_2O\ OH)_3$ were obtained and washed with DMF and CH_3CN three times.

Synthesis of TPP-MOL-Rh

A 5.6 mL CH_3CN suspension of TPP-MOL (46.8 μmol TPP) was stirred with 58 mg $[Rh(COD)Cl]_2$ (2.5 equiv. vs TPP) in CH_3CN at room temperature for 12 h and then washed three times using CH_3CN and CH_3OH. TPP-MOL-Rh was transferred into a N_2-filled glovebox for CD_3OD solvent exchange under N_2 atmosphere. The catalyst was dispersed in 5.6 mL CD_3OD for further PHIP experiments. ICP-OES analysis indicated that the Rh/TPP ratio of the load was 99.9%, allowing for quantitative determination of TPP-MOL-Rh.

Synthesis of TPP-MOL-Rh-P

A 2.8 mL CH_3CN suspension of TPP-MOL-Rh (23.4 μmol TPP) was stirred with 21 mg tris(4-methylphenyl)phosphine (CH_3-P, 3 equiv. vs TPP) in CH_3CN at room temperature for 24 h and then washed three times using CH_3CN and CH_3OH. The similar solvent exchange under N_2 atmosphere was performed for further PHIP test. ICP-OES analysis indicated that the Rh/TPP ratio of the load was ~73.5%, allowing for quantitative determination of TPP-MOL-Rh-P.

Synthesis of TPP-MOL-Rh-2PPh$_3$

A 2 mL CH_3CN suspension of TPP-MOL (16.7 μmol TPP) was stirred with 23 mg $Rh(PPh_3)_3Cl$, (1.5 equiv. vs TPP) in a mixed solvent of CH_3CN and THF at room temperature for 24 h and then washed with three times using CH_3CN, THF and CH_3OH. The similar solvent exchange under N_2 atmosphere was performed for further PHIP test. ICP-OES analysis indicated that the Rh/TPP ratio of the load was

~68.2%, allowing for quantitative determination of TPP-MOL-Rh-2PPh$_3$.

Synthesis of TPP-MOL-Rh-dppb

A 3.5 mL CH_3CN suspension of TPP-MOL (29.3 μmol TPP) was stirred with Rh(COD)(dppb)BF_4 (64 mg, 3 equiv. vs TPP) in CH_3CN at room temperature for 24 h and then washed three times using CH_3CN and CH_3OH. The similar solvent exchange under N_2 atmosphere was carried out for PHIP experiments. ICP-OES data indicated that the Rh/TPP ratio of the load was ~39.8%.

Synthesis of TPP-MOL-Rh-dhpb

In a N_2-filled glovebox, a 4 mL CH_3CN suspension of TPP-MOL (33.4 μmol TPP) was stirred with [Rh(COD)Cl]$_2$ (42 mg, 2.5 equiv. vs TPP) and bis(dicyclohexylphosphanyl)butane (dhpb, 76 mg, 5 equiv. vs TPP) in a mixed solvent of CH_2Cl_2 and CH_3CN at room temperature for 24 h and then washed with three times using CH_2Cl_2, CH_3CN and CH_3OH. The similar solvent exchange under N_2 atmosphere was performed for PHIP test. ICP-OES analysis showed that the Rh/TPP ratio of the load was ~22.5%, allowing for quantitative determination of TPP-MOL-Rh-dhpb.

4. PXRD simulation of TPP-MOL

The simulated PXRD pattern of TPP-MOL was obtained by a Matlab code using a CIF files as structural input (https://github.com/Wang-Group/2D-XRD-Modelling).[3] In Figure 2g of the manuscript, the peaks of PXRD pattern of TPP-MOL are not symmetrical Gaussian profile: a rapid increase of diffraction intensity on the low-angle side and a slow decrease on the high-angle side, which is a unique PXRD peak profile of the ultrathin material.[4]

5. Strategies for HET-PHIP

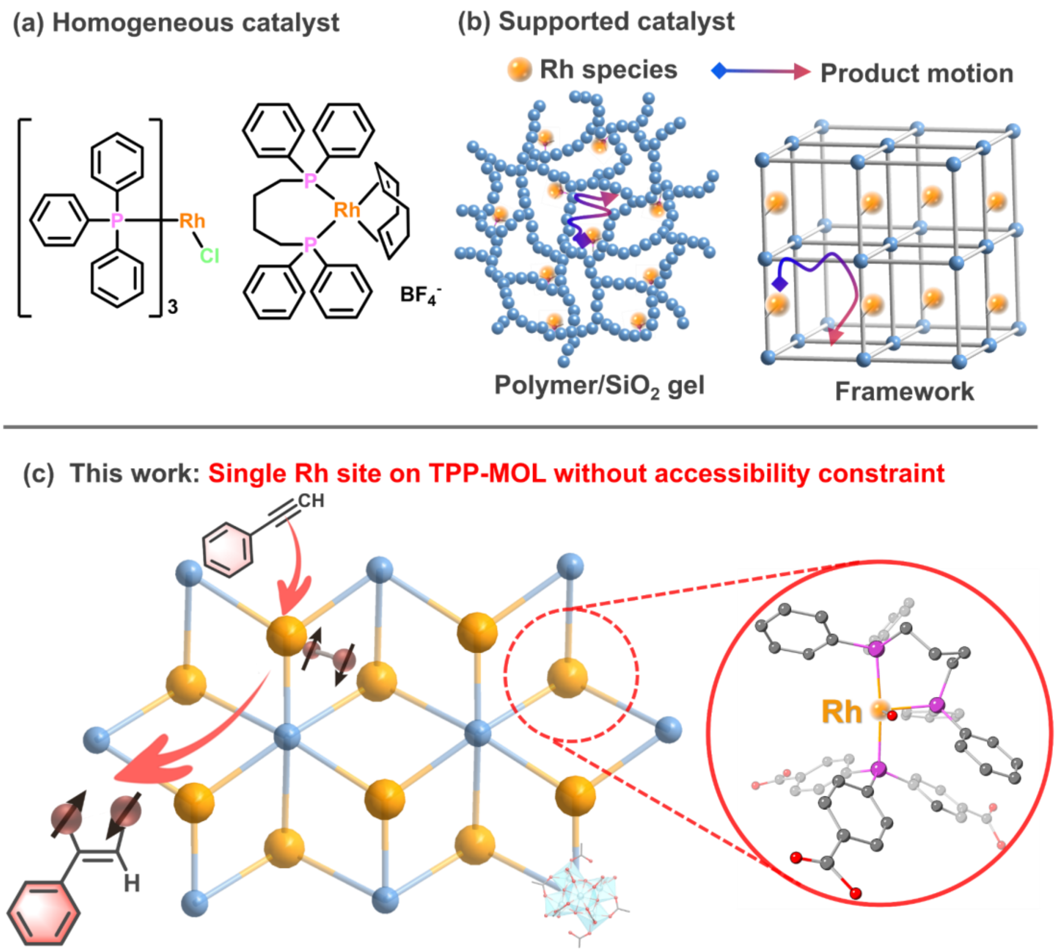

Figure S1 (a) Overview of known homogeneous catalysts and (b) Strategies for HET-PHIP. (c) Illustration of a single Rh site on TPP-MOL, highlighting its unrestricted accessibility.

6. Analysis of composition for TPP-MOL

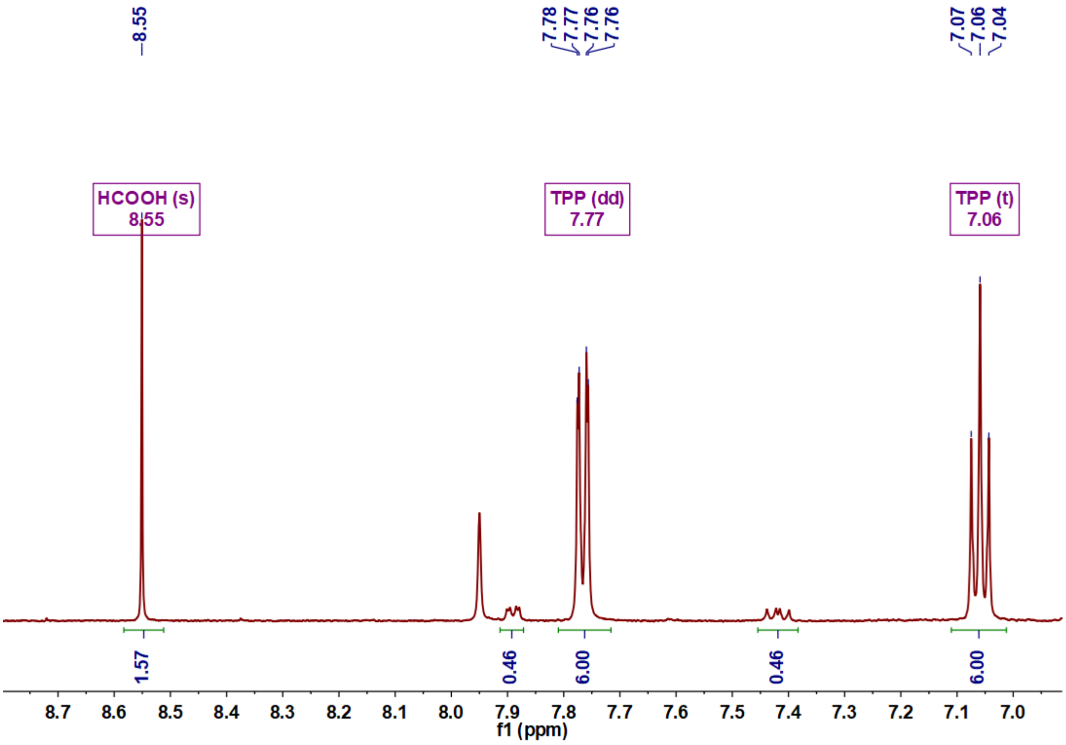

Figure S2 ^{1}H-NMR spectrum of the digested TPP-MOL by tetra-n-butylammonium fluoride (TBAF) in DMSO-d_6. n(HCO_2H) : n(BTB) = 1.6 : 1 was obtained from the integrations.

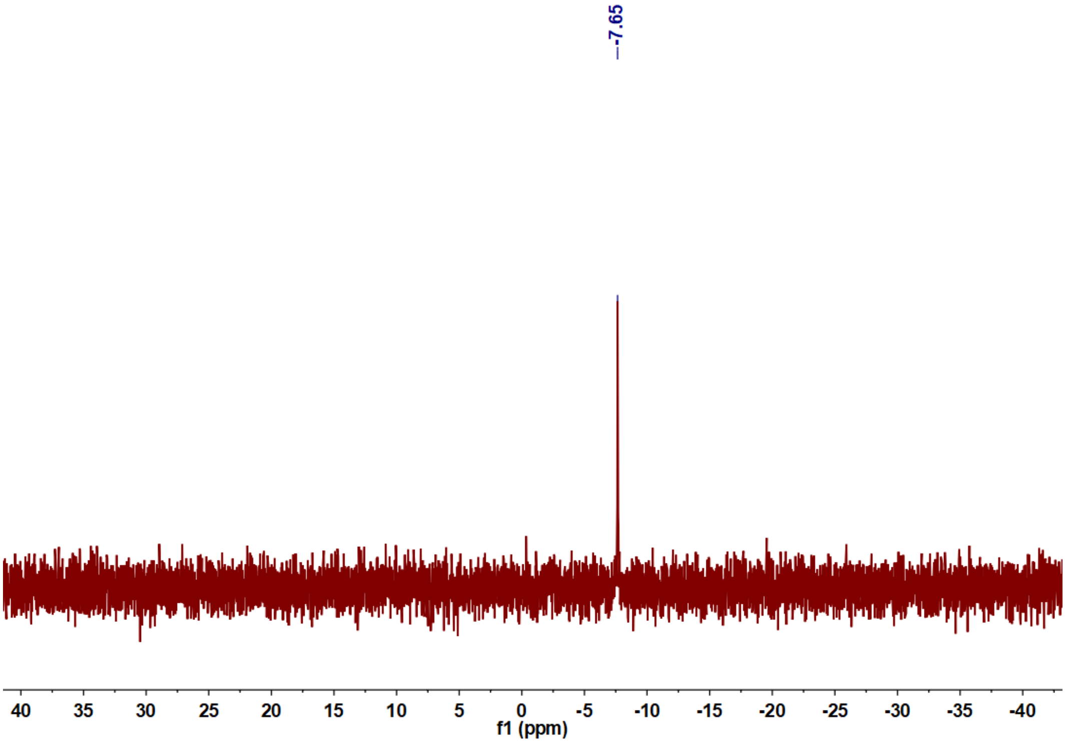

Figure S3 ^{31}P-NMR spectrum of the digested TPP-MOL by TBAF.

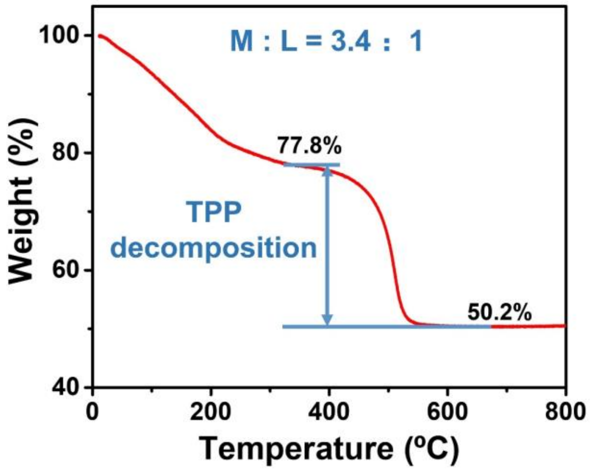

Figure S4 TGA analysis of TPP-MOL. The molar ratio of Hf : TPP is 3.4 : 1. A weight loss of 22.2% from RT to 400 ℃ was attributed to the loss of solvents and the decomposition of the coordinated $HCOO^-$ (~150 ℃ to 350 ℃). The subsequent loss of 27.6% from 400 ℃ to 580 ℃ corresponds to the decomposition of the TPP ligand.

7. Rh loading amount of MOL catalysts

The Rh:TPP ratio was calculated using the Rh:Hf ratio from ICP-OES. Initially, the Rh:Hf ratio was obtained from the ICP-OES data, then the Rh:TPP ratio could be inferred based on the Hf:TPP ratio of 3:1 in the TPP-MOL structure.

Table S1 The quantitative analysis of MOL catalysts using ICP-OES analysis.

Catalyst	Rh:TPP (ICP-OES)	Rh wt%
**TPP-MOL-Rh**	**99.9%**	**6.5**
**TPP-MOL-Rh-P**	**73.5%**	**4.5**
**TPP-MOL-Rh-2PPh$_3$**	**68.2%**	**4.2**
**TPP-MOL-Rh-dppb**	**39.8%**	**2.5**
**TPP-MOL-Rh-dhpb**	**22.5%**	**2.5**
**TPP-MOL-Rh-dppb-AR**	**39.7%**	**2.4**

8. Morphology and elemental mapping of MOL catalysts

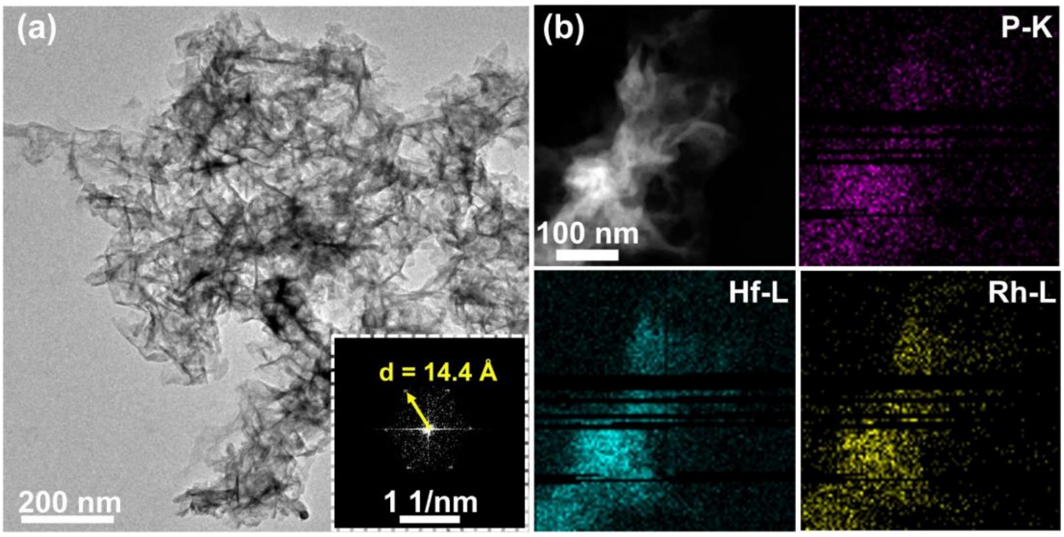

Figure S5 Characterization of TPP-MOL-Rh. (a) TEM image. The inset is the FFT pattern. (b) HADDF and EDX mapping images.

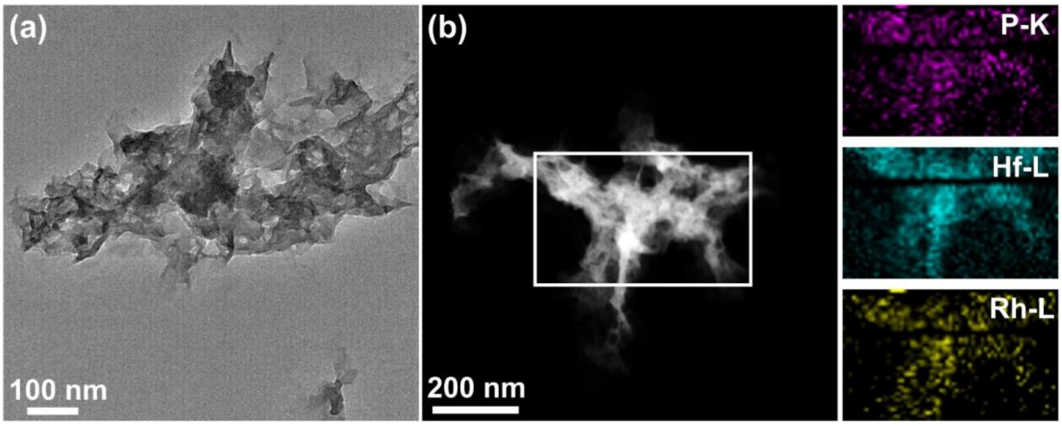

Figure S6 Characterization of TPP-MOL-Rh-P. (a) TEM image. (b) HADDF and EDX mapping images.

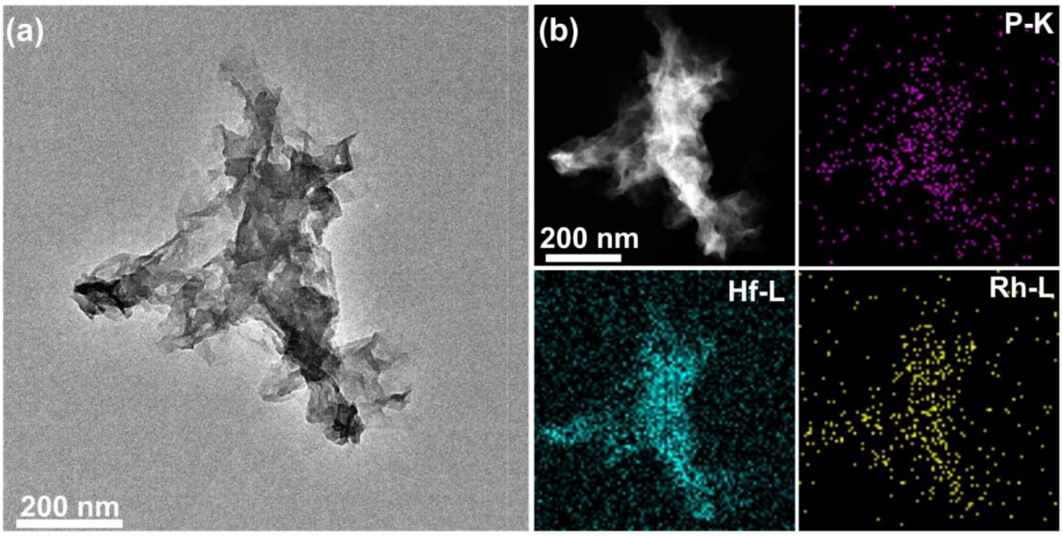

Figure S7 Characterization of TPP-MOL-Rh-2PPh3. (a) TEM image. (b) HADDF and EDX mapping images.

9. ¹H-NMR determination of multi(phosphine)-Rh sites

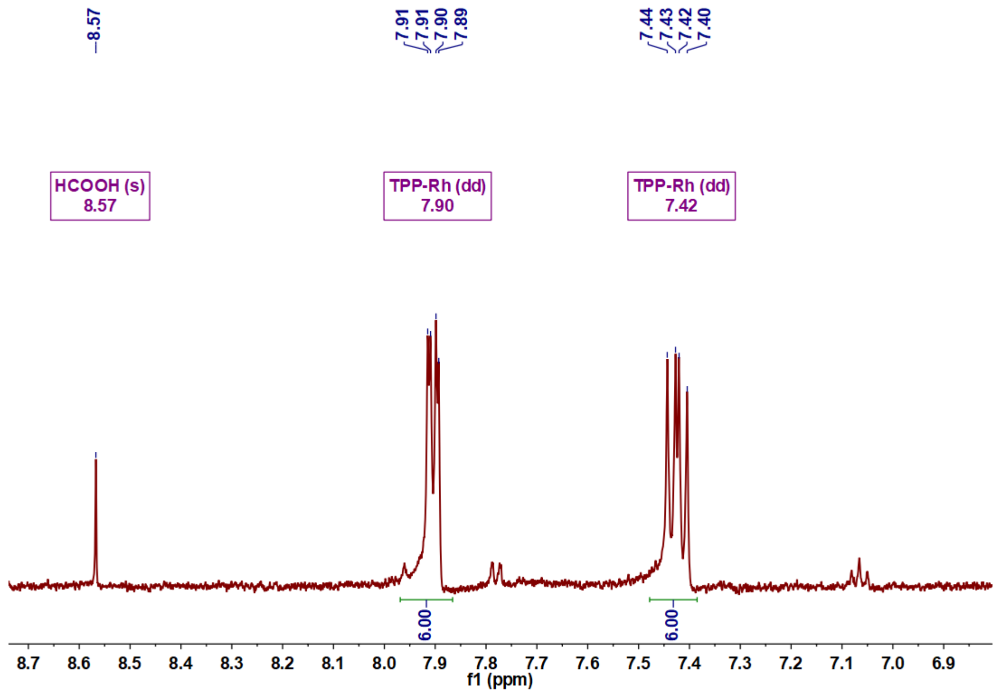

Figure S8 ¹H-NMR spectrum of the digested TPP-MOL-Rh by tetra-n-butylammonium fluoride (TBAF) in DMSO-d_6.

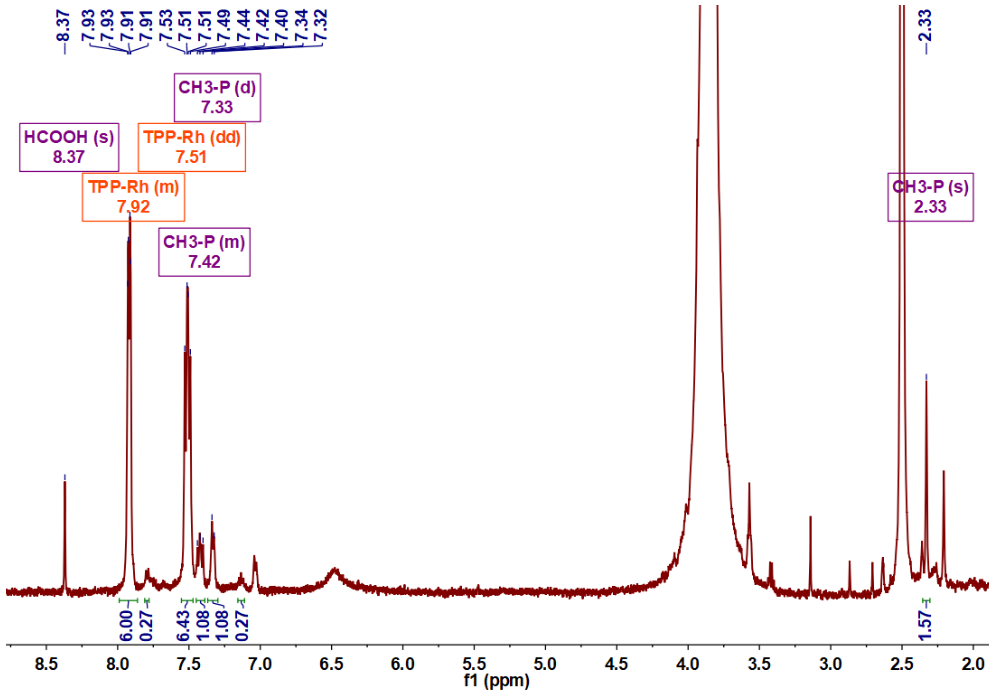

Figure S9 ¹H-NMR spectrum of the digested TPP-MOL-Rh-P. The dry sample was digested with 0.1 mL saturated K_3PO_4 solution in D_2O and then extracted with DMSO-d_6.

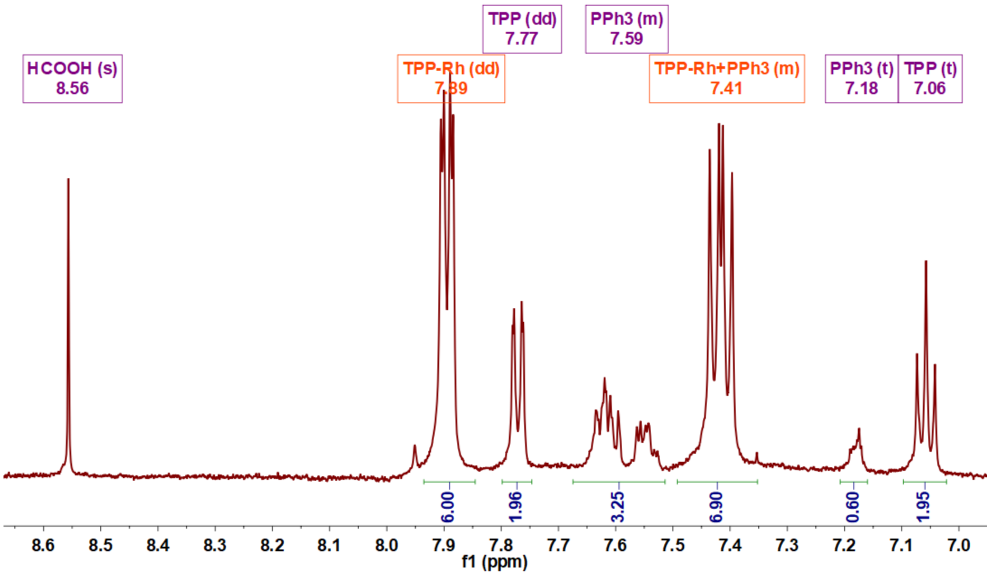

Figure S10 ^{1}H-NMR spectrum of the digested TPP-MOL-Rh-2PPh$_3$ by tetra-n-butylammonium fluoride (TBAF) in DMSO-d_6.

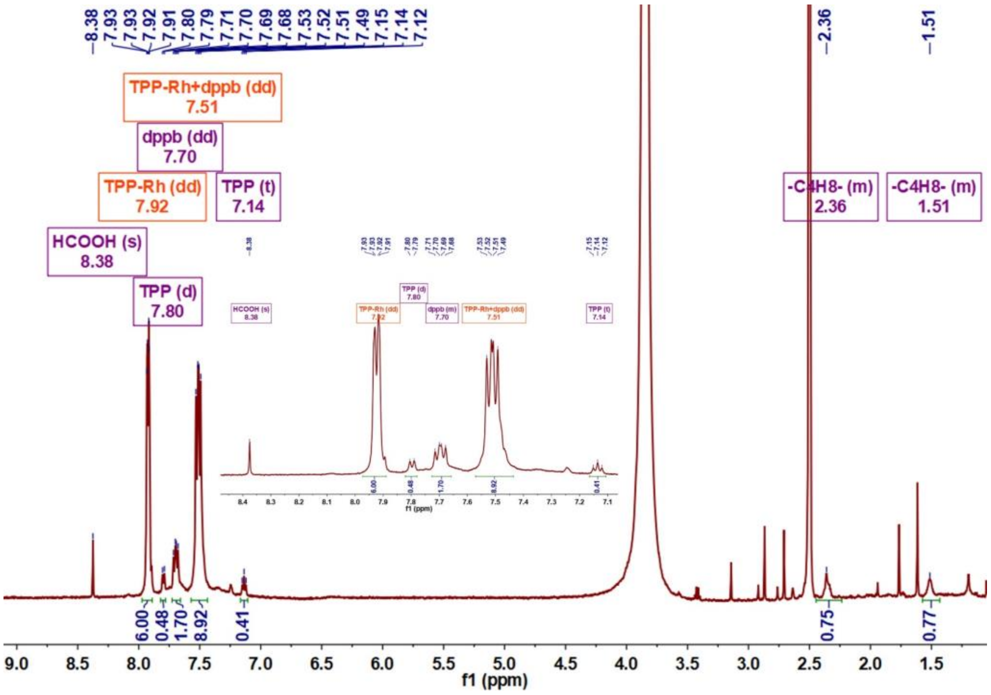

Figure S11 ^{1}H-NMR spectrum of the digested TPP-MOL-Rh-dppb. The dry sample was digested with 0.1 mL saturated K$_3$PO$_4$ solution in D$_2$O and then extracted with DMSO-d_6. The inset is the enlarged area of aromatic region.

10. XPS analysis of MOL catalysts

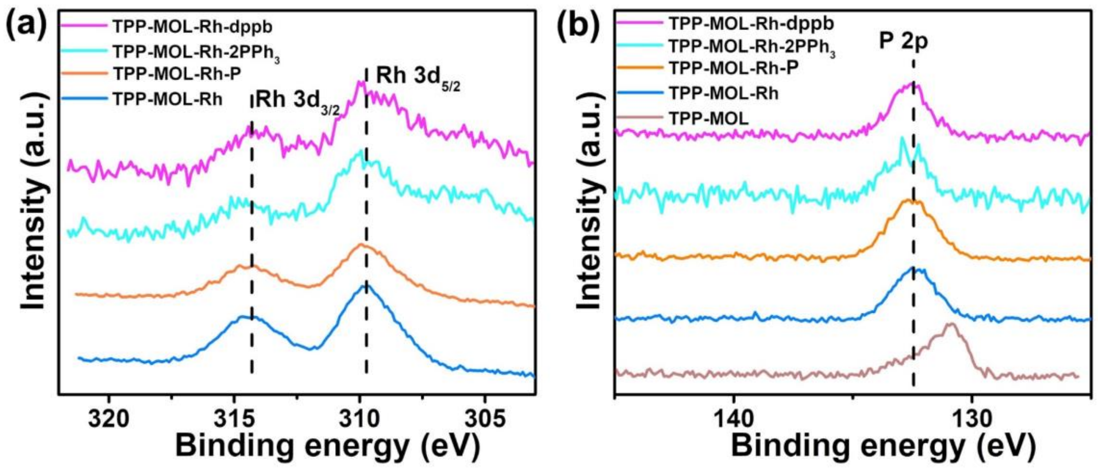

Figure S12 XPS spectra in (a) Rh 3d region of the four MOL catalysts and (b) P 2p region of the four MOL catalysts and uncoordinated TPP-MOL. The decrease in the intensity of XPS spectra corresponded to a reduction in Rh loading (from 99.9% to 22.5% with respect to TPP) for the four MOL catalysts.

11. Enrichment of parahydrogen and PASADENA ^{1}H-NMR experiments

Parahydrogen was enriched to 33% using our home-built parahydrogen generator, where normal hydrogen gas (n-H_2) is passed through a copper tube containing FeO(OH) catalyst. The temperature of copper tube and the catalyst is cooled by immersing it in liquid N_2 (77 K). The purity of 33% p-H_2 may fluctuate due to heat exchange.

Parahydrogen (96%) enrichment was generated by passing normal hydrogen gas (n-H_2) over a FeO(OH) catalyst in a closed-cycle cryostat operating at 35 K.

The spin quantum number I = 0 of parahydrogen cannot be observed by NMR, but the spin quantum number I = 1 of orthohydrogen can be obtained by NMR spectrometer. The formula for calculating the actual proportion of parahydrogen content (f%) in hydrogen is as follows[5]:

$$f\% = 1 - \frac{S_{p-H_2}}{S_{n-H_2}} * 75\%$$

Where $S_{n\text{-}H_2}$ is the integral area of ^{1}H NMR signal of normal hydrogen in NMR tube under a gas pressure at room temperature, and $S_{p\text{-}H_2}$ is the integral area of ^{1}H NMR signal of parahydrogen in an NMR tube under the same temperature and pressure.

In this paper, the concentration of p-H_2 was determined by 500 MHz Varian liquid NMR spectrometer (Agilent Technologies, Santa Clara, CA, USA), and the results are shown in Figure S13.

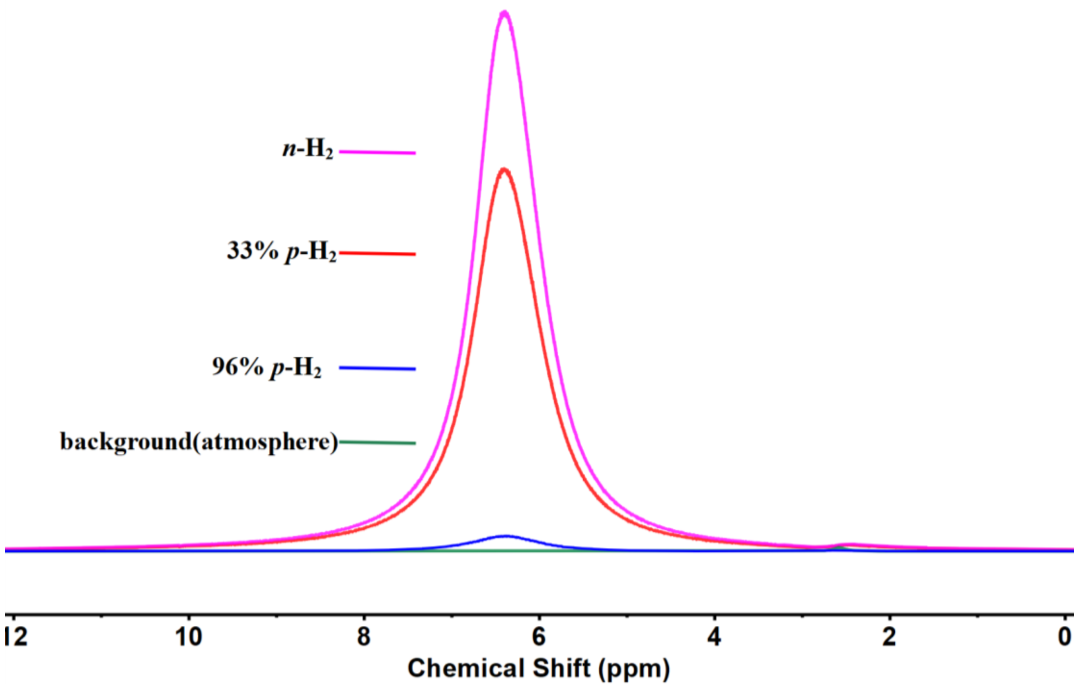

Figure S13 ^{1}H NMR spectra of NMR tubes filled with *n*-H_2 (pink), 33% *p*-H_2 (red) and 96% *p*-H_2 (blue) under 3 bar pressure at room temperature, and ^{1}H NMR spectra of NMR tube filled with air (green) (pw = 90°, d1 = 5, nt = 64).

The PASADENA procedure was employed for the PHIP test. In a glovebox, the TPP-MOL-Rh-dppb catalyst or other catalysts (0.44 mol% Rh or other Rh loading), substrate (0.182 mmol), CD_3OD (0.7 mL) were added into a medium-wall 5 mm NMR tube. The NMR tube was then transferred into a 500 MHz Varian liquid NMR spectrometer and heated to 50°C within 3 min. 33% or 96% *p*-H_2 was bubbled through the samples to 3 bar with a gas flow rate of 28 sccm. The hyperpolarized ^{1}H NMR spectra were acquired (nt = 1) using an array of $\pi/4$ radio frequency pulses. All spectra were normalized using peaks in aromatic region.

12. Calculation of signal enhancement and yield

The signal enhancement (SE) factor is calculated using the formula for the gas-liquid-solid phase from Igor V. Koptyug's report[6], as follows:

$$SE = \frac{S_{PHIP} - (S_{bef} * X)/2}{S_{bef} * X/n}$$

$$X = 1 - \frac{S_{aft}}{S_{bef}}$$

Where S_{PHIP} is the integral of the polarized styrene –CH or $–CH_2$ group signal in the spectra obtained with p-H_2, and n is the number of protons in the corresponding group. S_{bef} stands for the integral signal of the solution bubbling with normal H_2 (n-H_2) for 40 s under 3 bar pressure. S_{bef}*X represents the expected integral of the thermally polarized hydrogenation products. The observed PASADENA signal comprises the PHIP signal and half of the thermally polarized signal. S_{bef} is the corresponding signal intensity before termination of n-H_2 bubbling and S_{aft} is the integral of signal after introduction of n-H_2 at a flow rate of 28 sccm. SE of other substrates were calculated using the same formula.

In order to evaluate the levels of polarization achieved, it is essential to compare the SE obtained through experimentation (SE_{expt}) with the theoretical value (SE_{theor}). The polarization level and theoretical signal enhancement (SE_{theor}) multiplier is calculated as follows:

$$SE_{theor} = \frac{\lambda}{3}(4\chi_p - 1)\frac{2K_BT}{\gamma\hbar B_0}+1$$

$$P = \frac{SE_{expt}}{SE_{theor}} \times 100\%$$

Where T = 323 K, B_0 = 11.7, λ = 1, γ = 2.67x10^8 rad/T.s, and χ_p = 0.33 or 0.96 (the fraction of p-H_2 in the hydrogen gas). The SE_{theor} is 1441.1 and 12746.15 for PASADENA experiments with signal detection at 33% and 96% parahydrogen fraction, respectively. SE_{expt} is the polarization signal enhancement multiplier in the actual experiment.

The yield and turnover frequency (TOF, the number of catalytic reaction turnovers that occur per active site molecule per unit of time) were calculated based on the ^{1}H-NMR data of n-H_2 hydrogenation as follow:

$$\text{Yield \%} = \frac{\text{n}_{production}\,(\text{mol})}{\text{n}_{substrate}(\text{mol})} \times 100\%$$

$$\text{TOF} = \frac{Substrate\ (\text{mol}) \times \ \text{yield}\ (\%)}{\text{Loading Rh (mol)} \times t\ (min)}$$

13. PHIP activity of MOL heterogeneous catalysts using 33% p-H_2

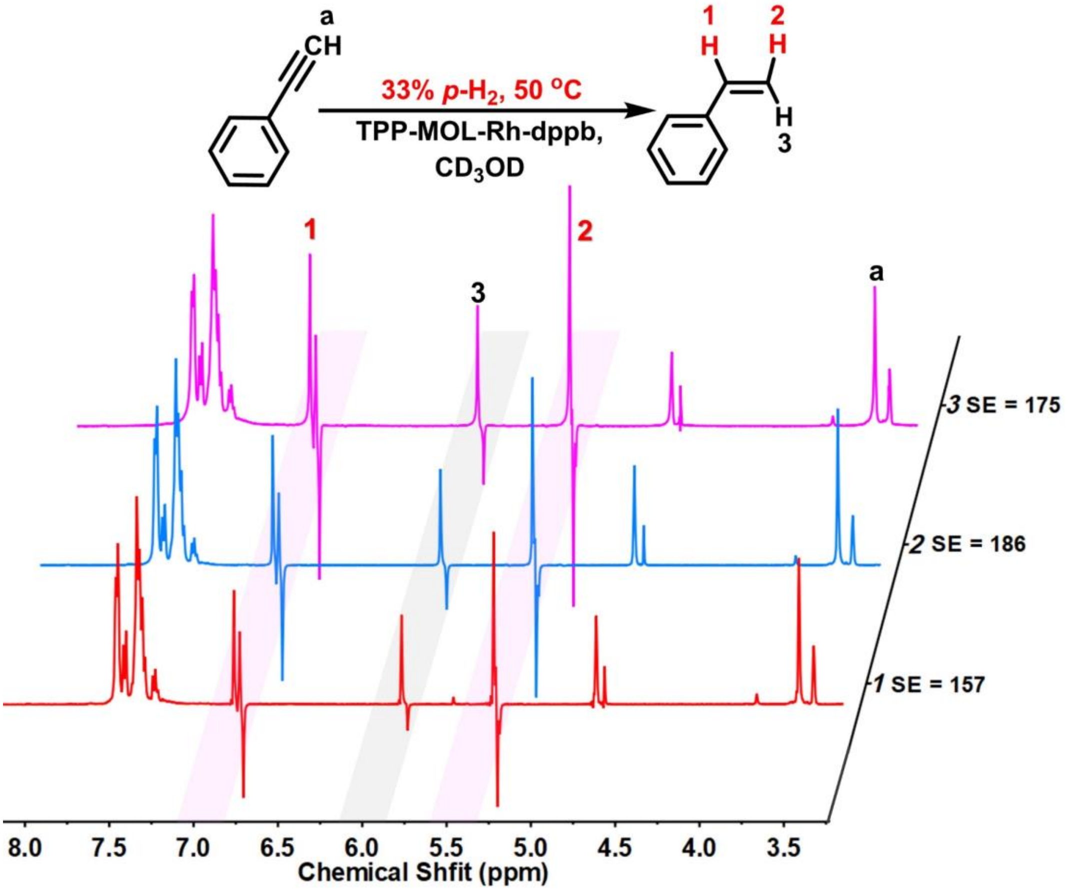

Figure S14 PASADENA ^{1}H-NMR spectra of TPP-MOL-Rh-dppb catalyst acquired during phenylacetylene hydrogenation with 33% p-H_2 for three repeated experiments.

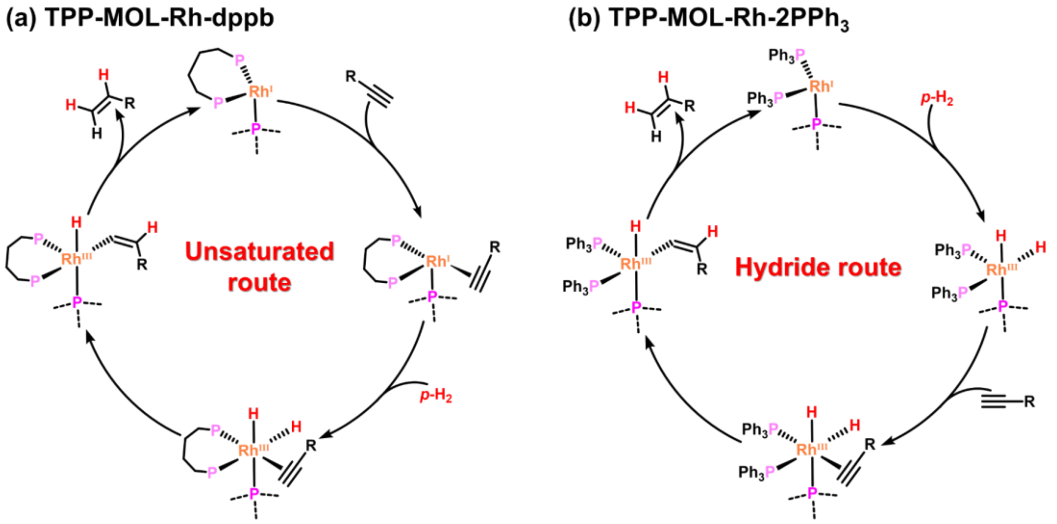

Figure S15 Proposed catalytic cycle for pairwise hydrogenation using (a) TPP-MOL-Rh-dppb and (b) TPP-MOL-Rh-2PPh$_3$ catalyst.[7]

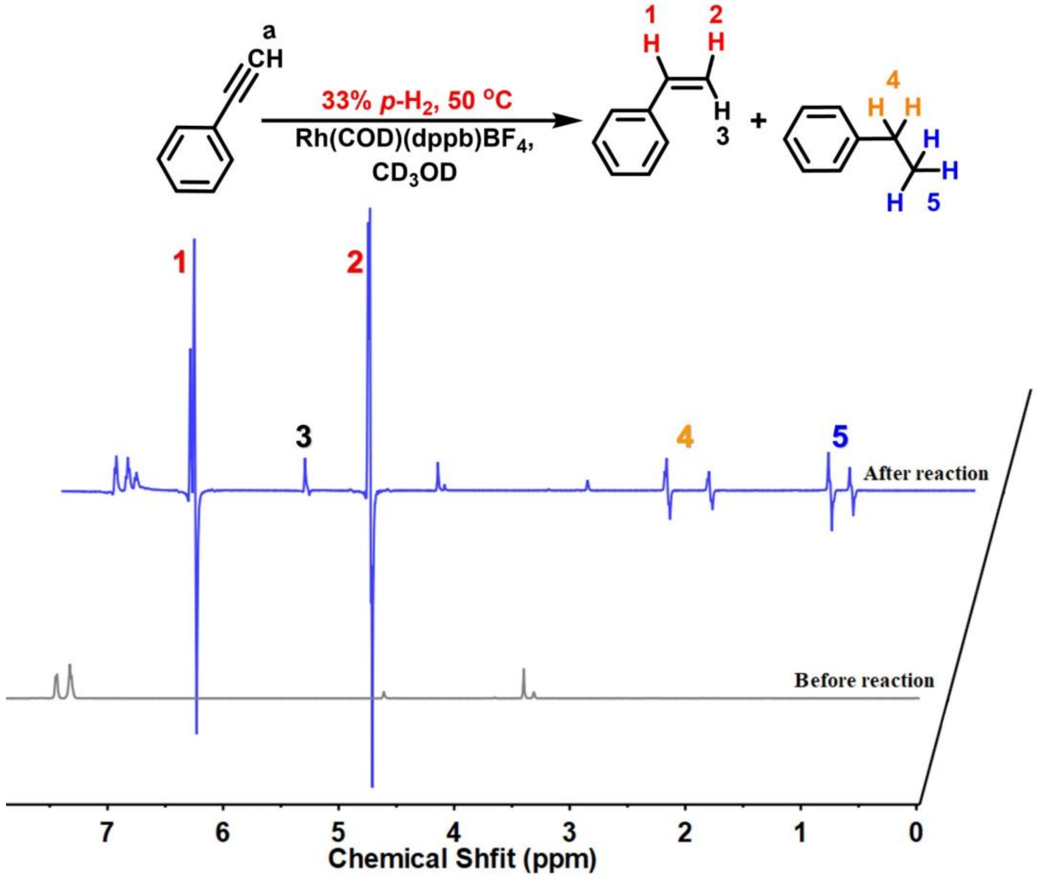

Figure S16 PASADENA ^{1}H-NMR spectra for phenylacetylene hydrogenation over homogeneous Rh(COD)(dppb)BF$_4$ catalyst using 33% *p*-H$_2$.

Table S2 PHIP activity over different catalysts.

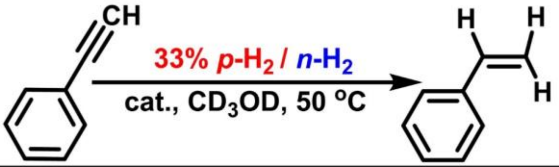

Entry	Catalyst	Rh loading (mol%)	3bar p-H_2[a]	n-H_2 (220 s)[b]	
			Signal Enhancement	Yield of $PhCHCH_2$ (%)	TOF (min^{-1})
1	TPP-MOL-Rh	0.44	55±17 (44; 67)[c]	1.5	0.9
2	TPP-MOL-Rh-P	0.44	89±21 (74; 104)	6.1	3.8
3	TPP-MOL-Rh-2PPh_3	0.44	88±3 (91; 86)	3.6	2.2
4	TPP-MOL-Rh-dppb	0.44	173±15 (157; 186; 175)	4.4	2.7
5	TPP-MOL-Rh-dhpb[d]	0.33	202	9.4	7.8
6	Rh(COD)(dppb)BF_4[e]	0.44	204	57.6	35.8

[a]Reaction conditions: MOL catalyst (0.44 mol% Rh), 0.182 mmol of PhCCH, 0.7 mL of CD_3OD, 50 °C, 3 bar p-H_2 (33%) with the flow rate of 28 sccm, other conditions as indicated. [b]The bubbled duration of n-H_2 was 220 s with the flow rate of 28 sccm. [c]SE factor calculated from repeated experiments. [d]TPP-MOL-Rh-dhpb with a Rh loading of 0.33 mol%. [e]Rh(COD)(dppb)BF_4 (0.44 mol% Rh) as homogeneous catalyst.

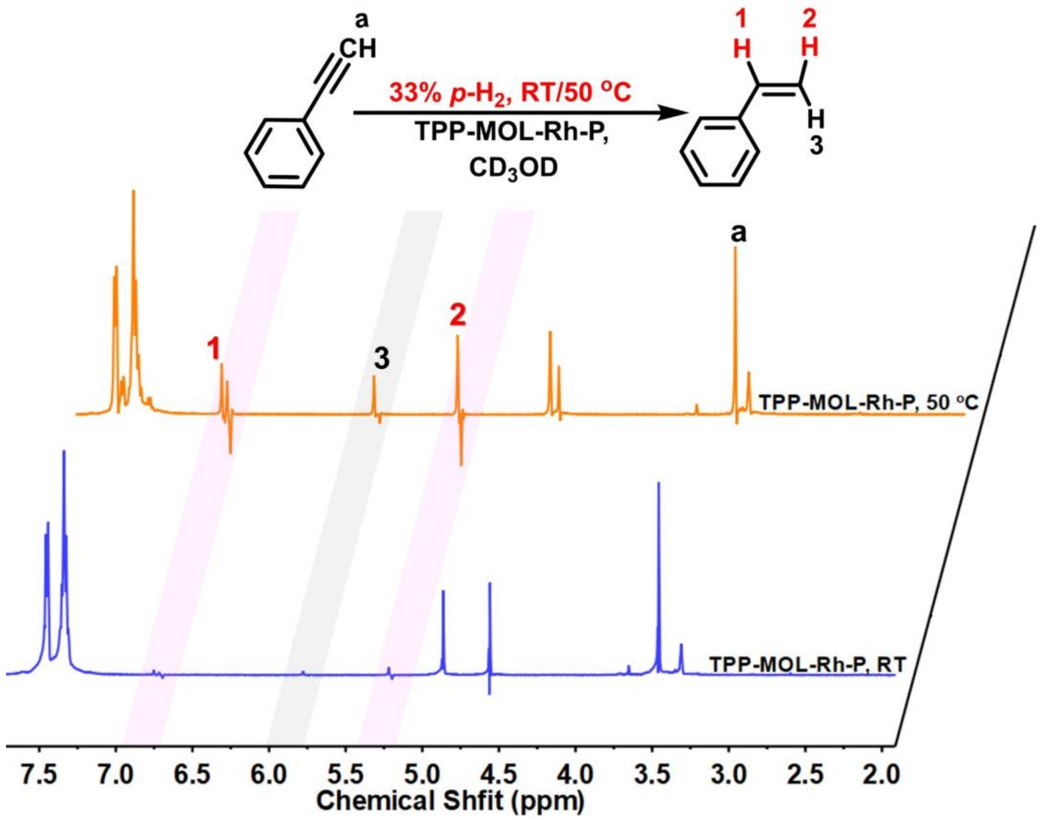

Figure S17 PASADENA ^{1}H-NMR spectra for phenylacetylene hydrogenation over TPP-MOL-Rh-P catalyst (0.44 mol% Rh) using 33% *p*-H_2 at room temperature and 50℃.

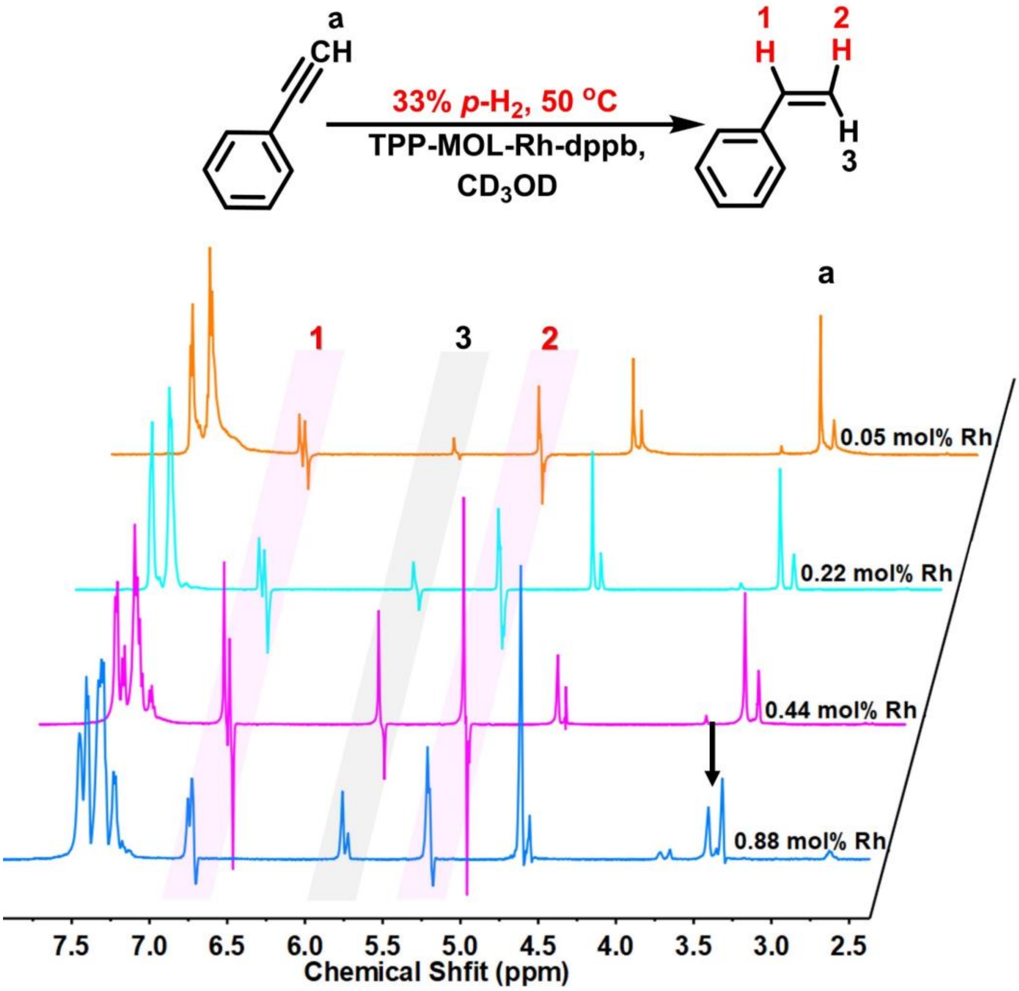

Figure S18 PASADENA ^{1}H-NMR spectra for phenylacetylene hydrogenation over TPP-MOL-Rh-dppb catalyst with various Rh loadings of 0.05, 0.22, 0.44, 0.88 mol% using 33% *p*-H_2. Higher Rh loadings of 0.88 mol% led to a noticeable increase in the consumption of phenylacetylene substrate (as indicated by the black arrow), implying that it was likely due to polymerization of phenylacetylene.

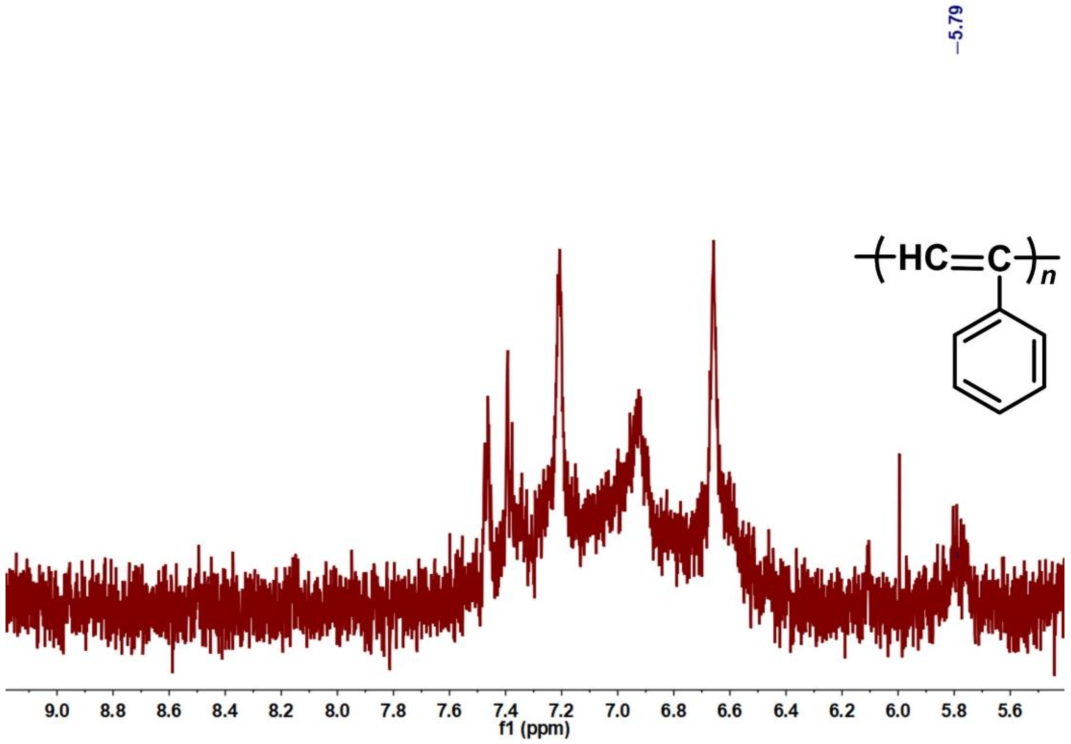

Figure S19 ^{1}H-NMR spectrum of polymerization products after separation. After phenylacetylene hydrogenation over TPP-MOL-Rh-dppb catalyst, the resulting yellow supernatant forms flocculent precipitates followed by settling. These precipitates are washed with CH_3OH and then dispersed in DMSO-d_6 for the detection of soluble oligomers by ^{1}H-NMR. The broad peak at δ 5.79 ppm corresponds to the proton peak of the ethynyl group in polyphenylacetylene.

Table S3 Summary of HET-PHIP catalysts used for generating gas-solid phase hyperpolarized products reported in the literature.

Entry	Category	Catalyst	Substrate (gas)	*p*-H_2[a]	Temp.	Procedure	SE	Ref.
1	Metal nanoparticle-based catalyst	$PtSn@mSiO_2$	propene	50%; 120 mL/min	200℃	ALTADENA	1213.8	8
					300℃	ALTADENA (theory)	3000	
2		Pt/CeO_2	propene	50%; 30 mL/min	300℃	ALTADENA	728	9
3		$Pd\text{-}In/Al_2O_3$	propyne	91%; 5.1 mL/s	200℃	ALTADENA	592	10
					400℃	ALTADENA (theory)	3400	
4		$Pd\text{-}Ag/Al_2O_3$	propyne	50%; 300 sccm	250℃	ALTADENA	900	11
5		1wt%Rh/TiO_2	cyclopropane	82%; 480 sccm	135℃	ALTADENA	480	12
6		1wt%Rh/TiO_2	1,3-butadiene	90.5%; 3.8 mL/s	130℃	PASADENA	200	13
7		$Pd\text{-}In/Al_2O_3$	propyne	87%; 3.8 mL/s	400℃	ALTADENA	107	14
		Pd/Al_2O_3		90.5%; 3.8 mL/s	300℃		3	
		$Pd\text{-}Ag/Al_2O_3$					13	
8		Cu/SiO_2	propyne	50%; 300 sccm	250℃	ALTADENA	~230	15
		$Cu\text{-}Pd_{0.003}/SiO_2$					~210	
9		$Pd_1\text{-}Sn_{0.5}/Al_2O_3$	1,3-butadienen	92.5%; 120 sccm	-	PASADENA	28	16
		$Pd_1\text{-}Sn_1/Al_2O_3$					51	
		$Pd_1\text{-}Sn_{1.5}/Al_2O_3$					62	
		$Pd_1\text{-}Sn_2/Al_2O_3$					100	
10		SI-Pd_1Au_1	propene	50%; 300 sccm	150℃	ALTADENA	~48	17
		IM-Pd_1Au_1					~10	
		DP-Pd_1Au_1					~15	
11		Rh/SP-S-1	propene	92%; 200 sccm	100℃	ALTADENA	49	18
		Rh/Nano-S-1					2.8	
		Rh@S-1					4.2	
12		1wt%Pt/SiO_2	propene	50%; 30 ccm	140℃	PASADENA	1219	19
13		hcp-Mo_2C	propyne	91.5%; 5.1 mL/s	400℃	ALTADENA	150	20
14		PVP-$PdAu/TiO_2$	1-butadiene	92%; 200 sccm	100℃	ALTADENA	440	21
15		Au/MWCNTs	1,3-butadiene	50%；5.1 mL/s	130℃	PASADENA	21	22
16		Ir/TiO_2	propane	50%；120 mL/min	150℃	ALTADENA	20	23
17		$Rh\text{-}In/SiO_2$	propyne	>98%; 600 sccm	200℃	ALTADENA	1420	24

Entry	Category	Catalyst	Substrate (gas)	p-H_2[a]	Temp.	Procedure	SE	Ref.
18	Immobilized metal-complex catalyst	3.8wt%Ir-PPh_2-SiO_2	propyne	90%; 2.2 mL/s	120℃	PASADENA	840	25
		3.4%wt%Rh-PPh_2-SiO_2					680	
19		V(═O)$(Mes)_3$/SiO_2 (VCAT/SiO_2)	propyne	80-90%; 2.1 sccs	500℃	ALTADENA	1300	26
20		Ir/SiO_2^P	propyne	50%; 3.4 sccs	120℃	PASADENA	500	27
						ALTADENA	300	
21		Ir-Py-TiO_2	propene	99.995%; 3.4 mL/s	100℃	PASADENA	220	28
		Ir-N-TiO_2			120℃		134	
22		Ir-PPh_2-SiO_2	propyne	50%; 4.3 mL/s	80℃	ALTADENA	400±100	29
23		Rh-PPh_2-SiO_2	propylene	50%	150℃	ALTADENA	180	30
24		IRMOF-3-SI-Au	propene	50%; 300 mL/min	130℃	ALTADENA	16	31

[a]The isotopic composition of H_2 and the method of H_2 introduction.

Table S4 Summary of HET-PHIP catalysts used for generating gas-liquid-solid phase hyperpolarized products reported in the literature.

<table>
<tr><th>Entry</th><th>Category</th><th>Catalyst</th><th>Substrate (liquid)</th><th>p-H_2[a]</th><th>Temp.</th><th>Procedure</th><th>SE</th><th>Ref.</th></tr>
<tr><td rowspan="6">1</td><td rowspan="14">Metal nanoparticle-based catalyst</td><td>Pd OCT-27</td><td rowspan="6">2-methyl-3-butyn-2-ol</td><td rowspan="6">50%; 70 mL/min</td><td>100℃</td><td rowspan="6">PASADENA</td><td>~17</td><td rowspan="6">32</td></tr>
<tr><td>Pd OCT-27</td><td rowspan="5">80℃</td><td>~8</td></tr>
<tr><td>Pd OCT-17</td><td>~4</td></tr>
<tr><td>Pd OCT-19</td><td>~4.8</td></tr>
<tr><td>Pd OCT-23</td><td>~5.5</td></tr>
<tr><td>Pd OCT-34</td><td>12</td></tr>
<tr><td rowspan="2">2</td><td>10wt%Rh/TiO_2</td><td>3-phenyl-1-propyne</td><td>50%; 6.1 bar</td><td>RT</td><td>PASADENA</td><td><20</td><td rowspan="2">6</td></tr>
<tr><td>4wt%Rh/TiO_2</td><td>phenylacetylene</td><td>50%; 6.1 bar</td><td>RT</td><td>PASADENA</td><td>~9</td></tr>
<tr><td>3</td><td>0.97wt%Rh/TiO_2</td><td>vinyl [1-^{13}C] acetate</td><td>89%; 140 sccm</td><td>85℃</td><td>ALTADENA</td><td>168</td><td>33</td></tr>
<tr><td rowspan="2">4</td><td rowspan="2">Pt_3Sn@mSiO_2</td><td rowspan="2">allyl acetate</td><td rowspan="2">99%; 2 mL/min</td><td>100℃</td><td>ALTADENA</td><td>212</td><td rowspan="2">34</td></tr>
<tr><td>80℃</td><td>ALTADENA</td><td>626</td></tr>
<tr><td>5</td><td>0.5wt%Rh/TiO_2</td><td>allyl acetate</td><td>50%; 100 mL/min</td><td>80℃</td><td>ALTADENA</td><td>99</td><td>35</td></tr>
<tr><td>6</td><td>1wt%Rh/TiO_2</td><td>methylcyclobutane</td><td>50%; 0.7 mL/s</td><td>100℃</td><td>ALTADENA</td><td>38</td><td>36</td></tr>
<tr><td>7</td><td>Rh/TiO_2</td><td>allyl acetate</td><td>50%; 14 mL/s</td><td>90℃</td><td>PASADENA</td><td>20</td><td>37</td></tr>
<tr><td>8</td><td>MSA-Pt NPs</td><td>(Z)-stilbene</td><td>51%; 3 bar</td><td>RT</td><td>ALTADENA</td><td>149</td><td>38</td></tr>
<tr><td>9</td><td>GSH@Pt</td><td>hydroxyethyl acrylate</td><td>95%; 5 bar</td><td>80℃</td><td>ALTADENA</td><td>50</td><td>39</td></tr>
<tr><td colspan="9"></td></tr>
<tr><td>This work</td><td>Immobilized metal-complex catalyst</td><td>TPP-MOL-Rh-dppb</td><td>phenylacetylene</td><td>96%; 3 bar</td><td>50℃</td><td>PASADENA</td><td>2404±200</td><td></td></tr>
</table>

[a]The isotopic composition of H_2 and the method of H_2 introduction.

14. Structure and HET-PHIP activity of TPP-MOL-Rh-dhpb

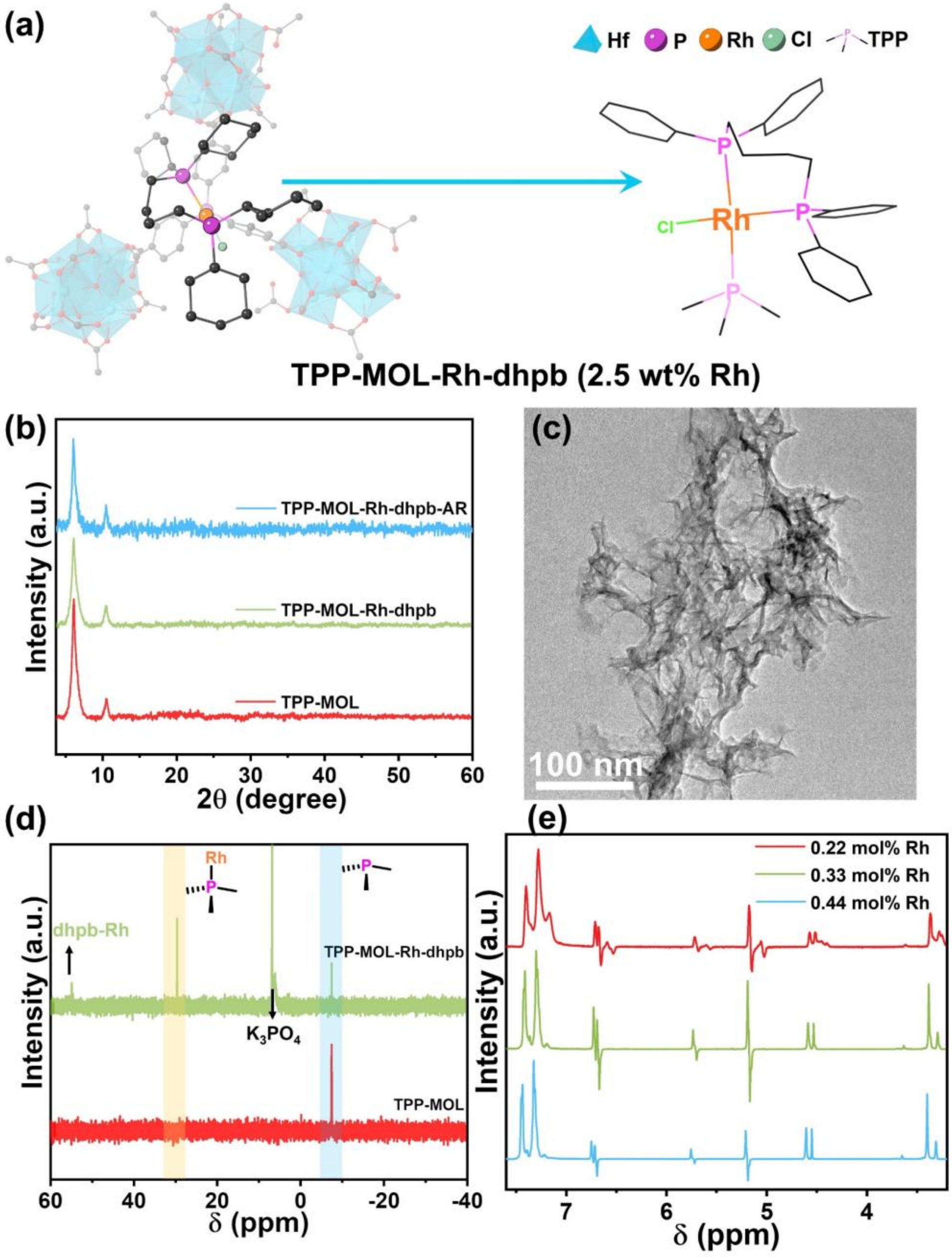

Figure S20 (a) Structure of TPP-MOL-Rh-dhpb. (b) PXRD patterns of TPP-MOL and TPP-MOL-Rh-dhpb before and after reaction. (c) TEM image of TPP-MOL-Rh-dhpb. (d) ^{31}P-NMR spectra of the digested of samples by the with 0.1 mL saturated K_3PO_4 solution in D_2O and then extracted with DMSO-d_6. (e) PASADENA ^{1}H-NMR spectra for phenylacetylene hydrogenation over TPP-MOL-Rh-dhpb catalyst with various Rh loading of 0.22, 0.33, 0.44 mol% using 33% p-H_2.

15. PHIP activity of TPP-MOL-Rh-dppb catalyst using 96% *p*-H_2

PASADENA ^{1}H-NMR experiments were performed using the TPP-MOL-Rh-dppb catalyst (Rh loading: 0.44 mol%, 0.30 mol% and 0.11 mol%) with 96% *p*-H_2 (3 bar), and the phenylacetylene (0.182 mmol) amounts were kept consistent.

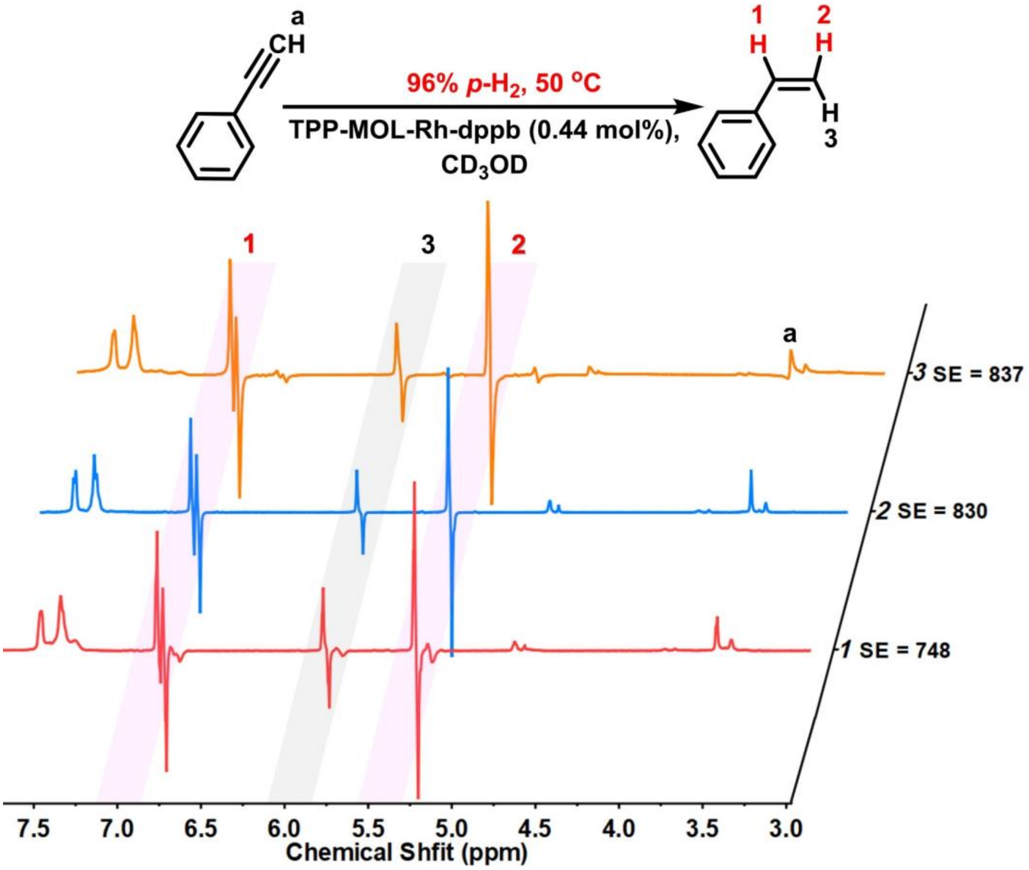

Figure S21 PASADENA ^{1}H-NMR spectra of TPP-MOL-Rh-dppb catalyst (0.44 mol% Rh) acquired during phenylacetylene hydrogenation with 96% *p*-H_2 for three repeated experiments.

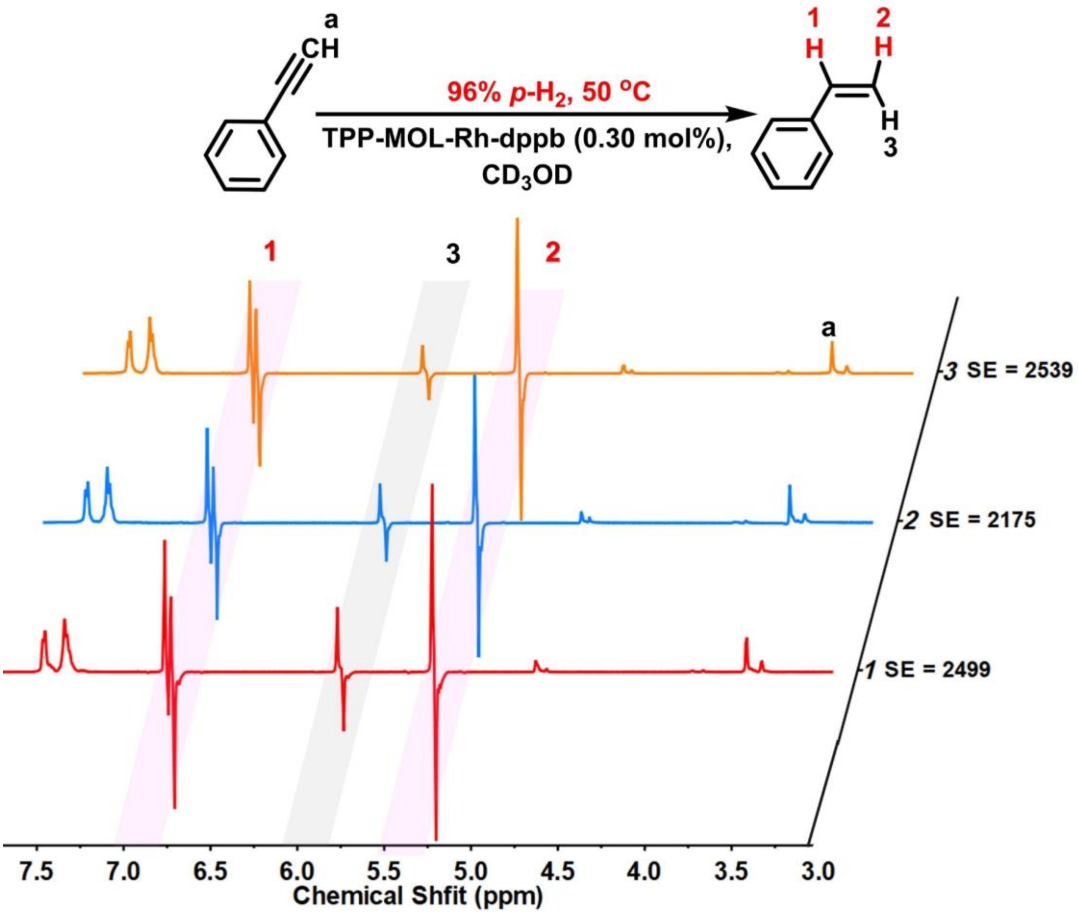

Figure S22 PASADENA ^{1}H-NMR spectra of TPP-MOL-Rh-dppb catalyst (0.30 mol% Rh) acquired during phenylacetylene hydrogenation with 96% p-H_2 for three repeated experiments.

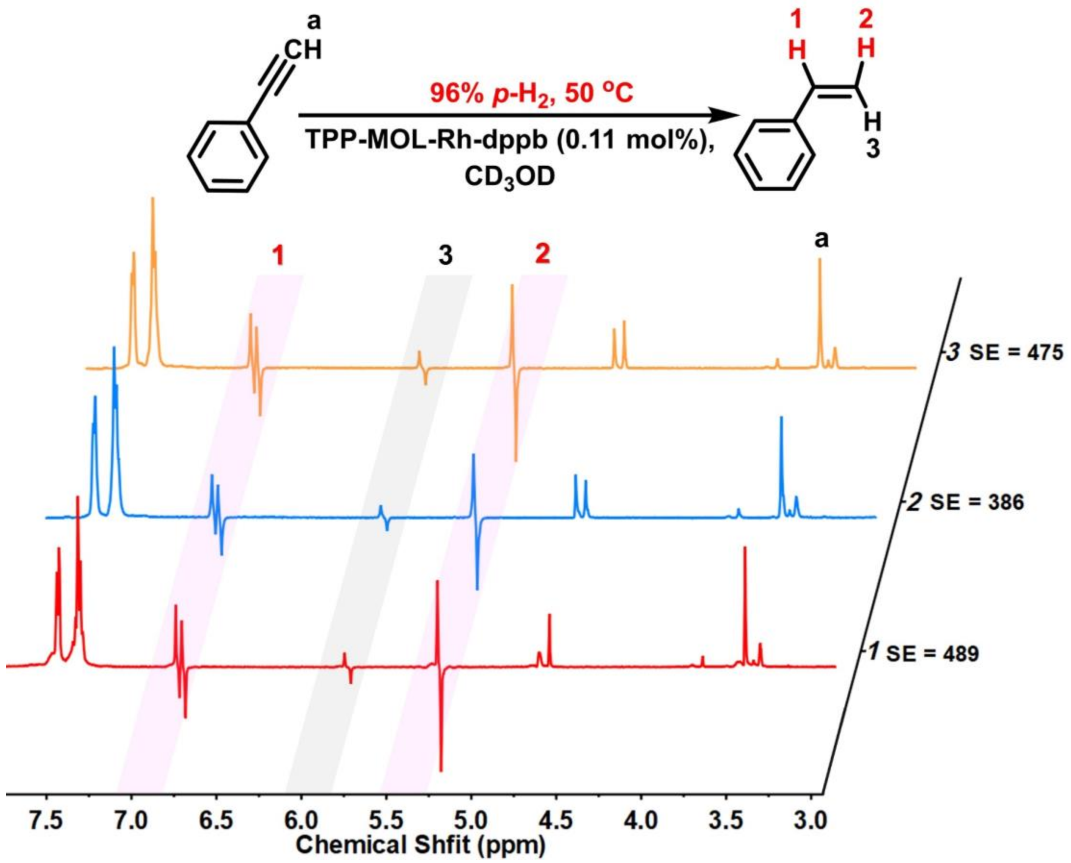

Figure S23 PASADENA ^{1}H-NMR spectra of TPP-MOL-Rh-dppb catalyst (0.11 mol% Rh) acquired during phenylacetylene hydrogenation with 96% *p*-H_2 for three repeated experiments.

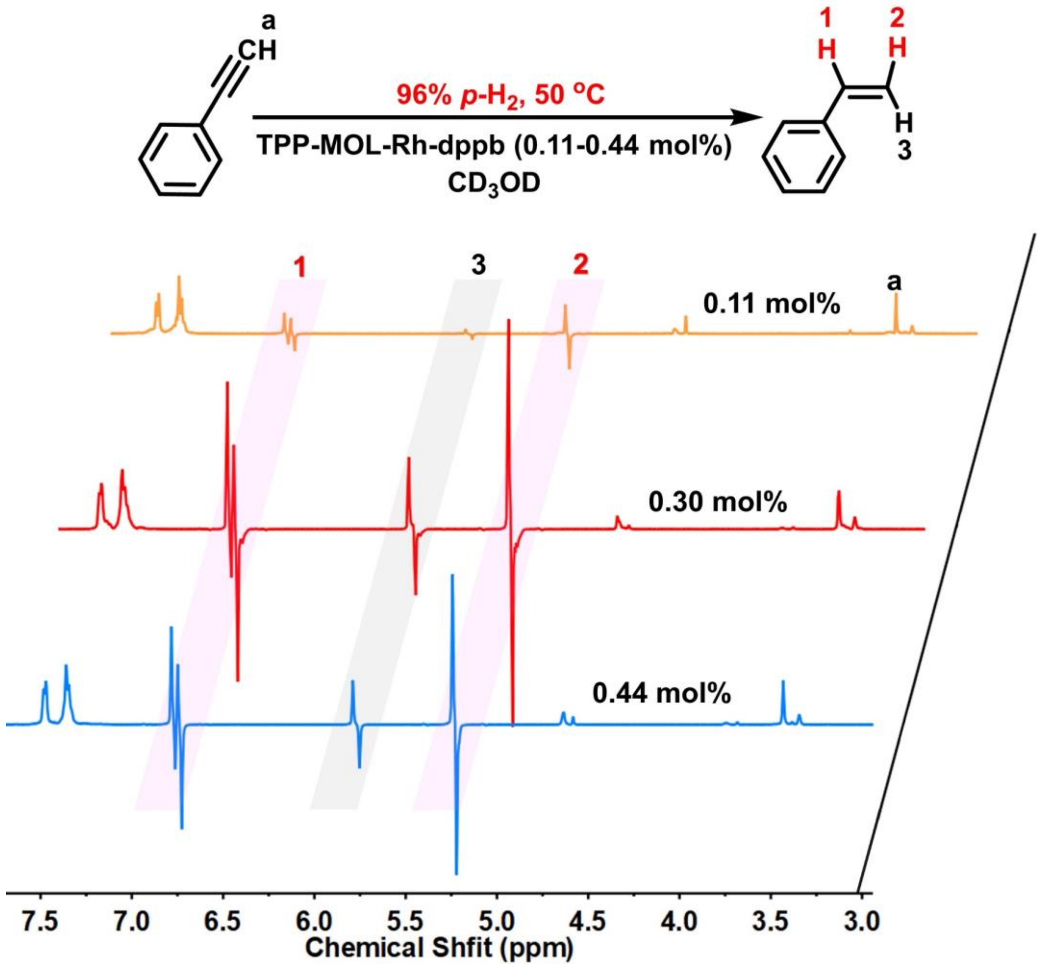

Figure S24 Summary of normalized PASADENA ^{1}H-NMR spectra using 96% p-H_2: TPP-MOL-Rh-dppb catalysts with different Rh loading of 0.44 mol%, 0.30 mol% and 0.11 mol%.

Table S5 HET-PHIP activity for TPP-MOL-Rh-dppb catalysts with different Rh loading using 96% p-H_2.

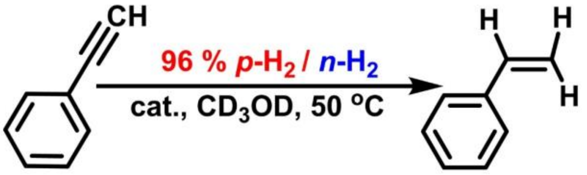

Entry	Catalyst	Rh loading (mol%)	3bar p-H_2 [a]		n-H_2 (220 s) [b]
			Signal Enhancement	Polarization (%)	Yield of $PhCHCH_2$ (%)
1	TPP-MOL-Rh-dppb	0.44	805±49 (748; 830; 837) [c]	6.3±0.4 (5.9; 6.5; 6.6)	4.4
2	TPP-MOL-Rh-dppb	0.30	2404±200 (2499; 2175; 2539)	18.9±1.5 (19.6; 17.1; 19.9)	1.8
3	TPP-MOL-Rh-dppb	0.11	450±56 (489; 386; 475)	3.5±0.4 (3.8; 3.0; 3.7)	1.4

[a]Reaction conditions: MOL catalyst (0.11-0.44 mol% Rh), 0.182 mmol of PhCCH, 0.7 mL of CD_3OD, 50°C, 3 bar p-H_2 (96%) with the flow rate of 28 sccm, other conditions as indicated. [b]The bubbled duration of n-H_2 was 220 s with the flow rate of 28 sccm. [c]SE factor and polarization (%) calculated form three repeated experiments.

Table S6 The signal enhancement factor and polarization (%) of ^1H improved by SABRE method were summarized in the literature.

Entry	Substrate	SE	Polarization	Catalyst	Ref.
1	Citronellol	200	0.65%	[IrCl(COD)(IMes)]	40
2	Pyrazine	1900	0.04%	[IrCl(COD)(IMes)]	41
3	Nicotinamide	620	0.21%	[IrCl(COD)(IMes)]	42
4	Acetonitrile	500	1.6%	Ir-IMes@NU-1000	43
5	3,5-Dichloropyridine	4350	14%	[IrCl(COD)(IMes)]	44
6	4,6-d_2-Nicotinate	100000	50%	[IrCl(COD)(d_{22}-IMes)]	45

Table S7 Summary of molecules that can be polarized by PHIP and SABRE methods reported in the literature.

**PHIP**		**SABRE**	
Vinyl pyruvate	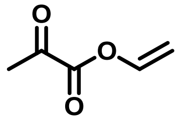	Acetonitrile	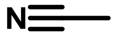
Phenylacetylene	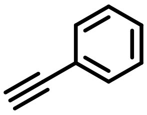	Pyridine	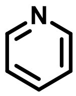
2-Methyl-3-butyn-2-ol	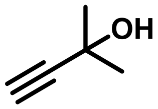	Pyruvate	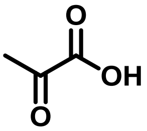
Propene	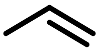	Amino acids	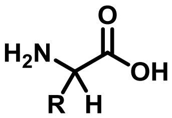
Hydroxyethyl acrylate	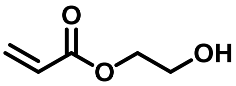	Imidazole	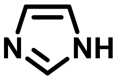

16. Heterogeneity test of MOL catalysts using 33% p-H_2

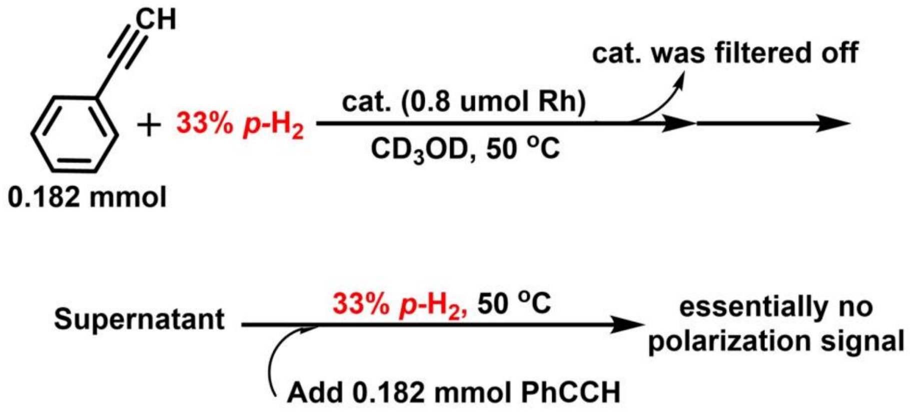

Figure S25 Testing the heterogeneity of MOL catalysts via a hot filtration test. The separated supernatant after the hydrogenation reaction as a catalyst to further react with fresh phenylacetylene addition using 33% p-H_2.

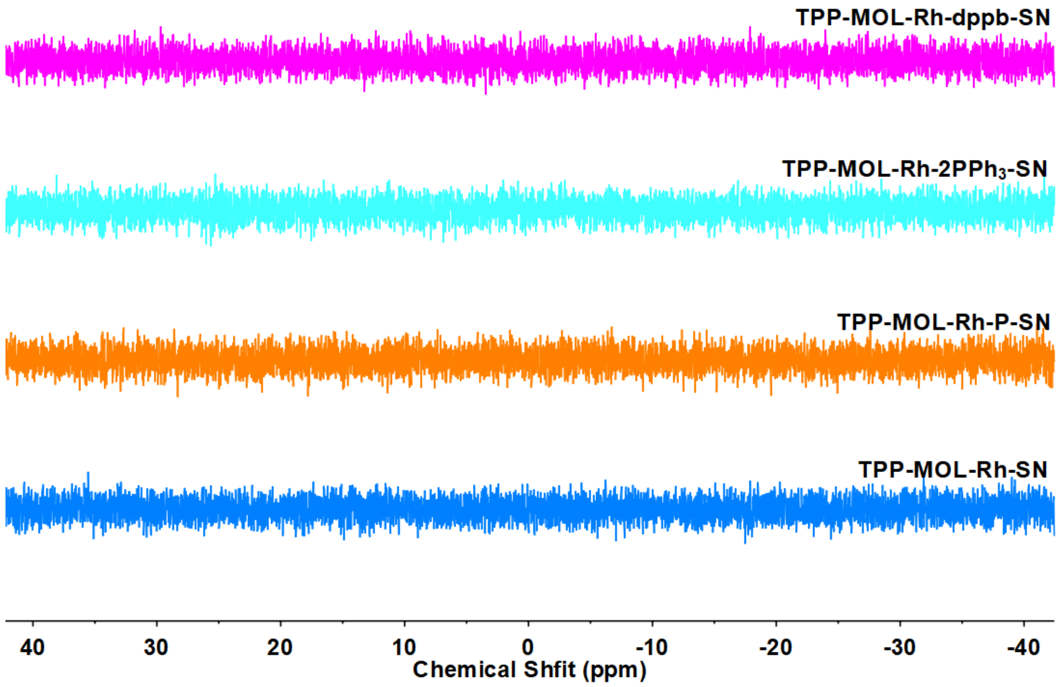

Figure S26 ^{31}P-NMR spectra of the separated supernatant for each catalyst after the hydrogenation. The spectra were detected with the same the number of scans (nt = 200).

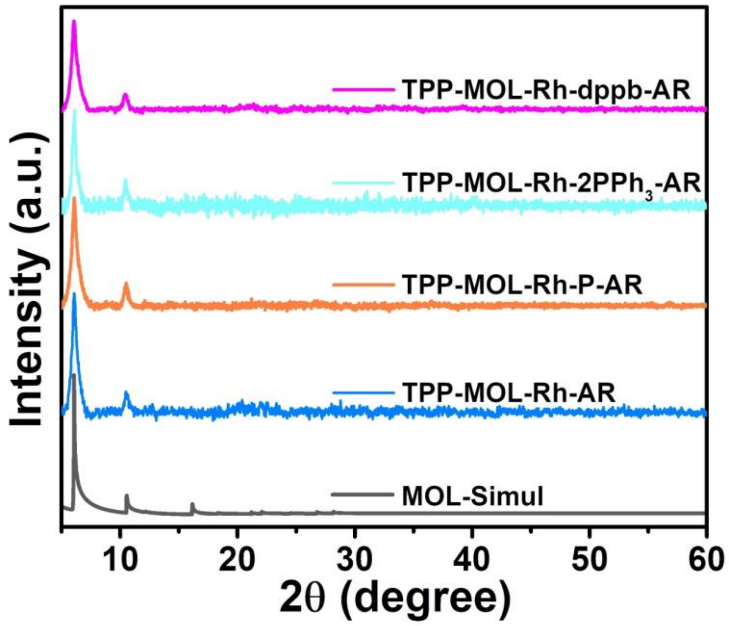

Figure S27 PXRD patterns of the recovered MOL catalysts after p-H_2 hydrogenation.

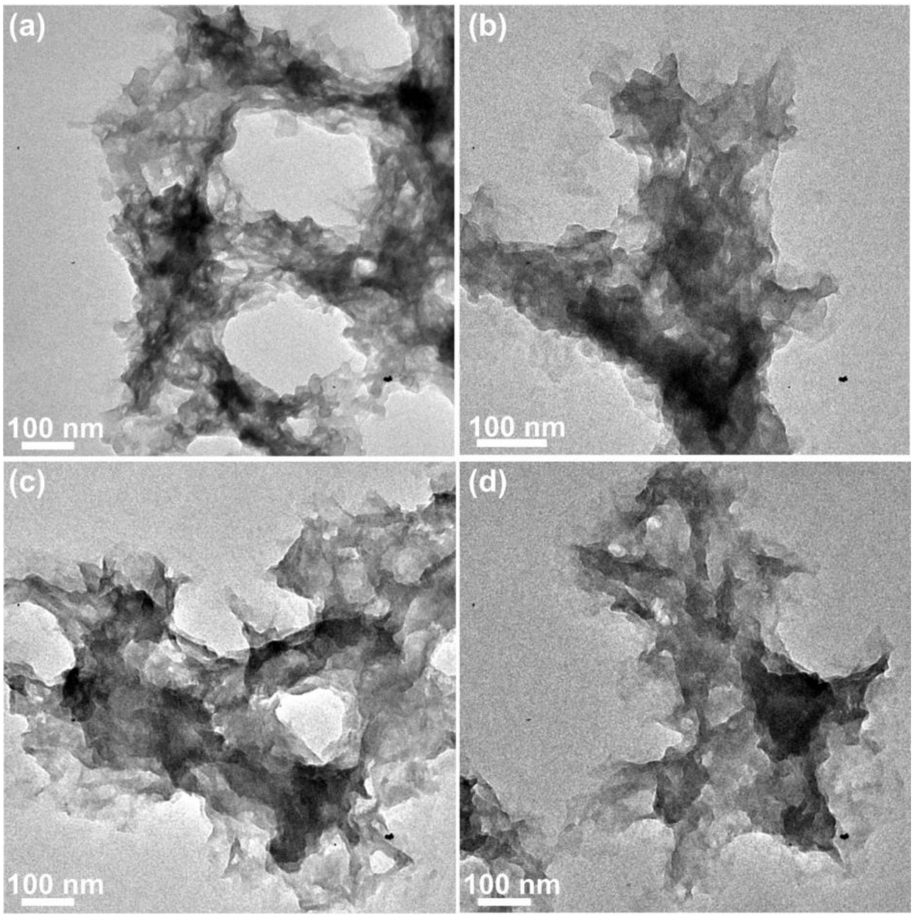

Figure S28 TEM images of (a)TPP-MOL-Rh, (b) TPP-MOL-Rh-P, (c) TPP-MOL-$2PPh_3$ and (d) TPP-MOL-Rh-dppb catalyst after the hydrogenation.

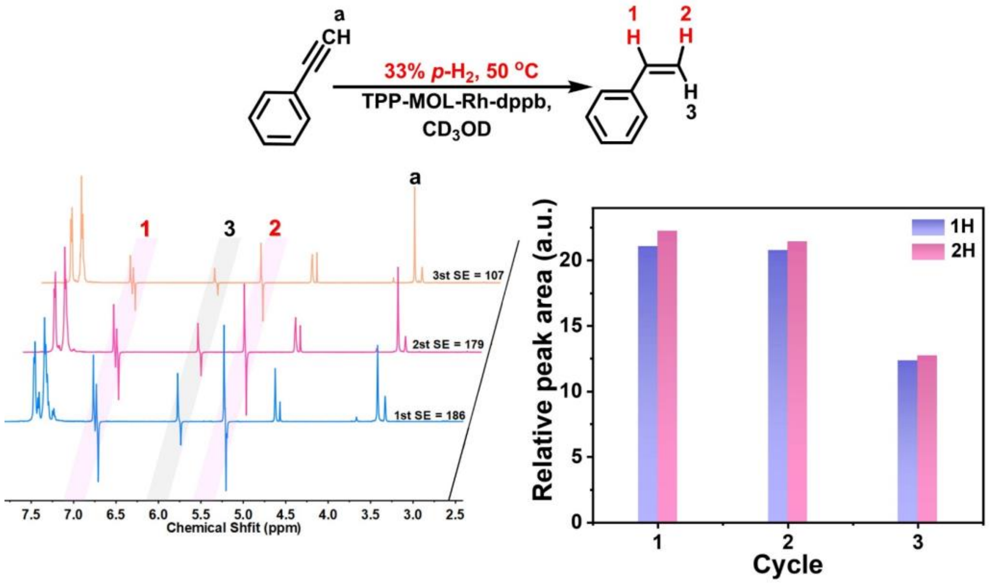

Figure S29 Recycling of TPP-MOL-Rh-dppb catalyst for PHIP test using 33% p-H_2. In a N_2-filled glovebox, TPP-MOL-Rh-dppb (0.44 mol% Rh), phenylacetylene (20 μl, 0.182 mmol), CD_3OD (0.7 mL) were added into an NMR tube. After p-H_2 hydrogenation, TPP-MOL-Rh-dppb catalyst was separated by centrifugation. The recovered catalyst was then washed with CD_3OD three times to be used for subsequent catalytic cycles under identical condition. The spectra are normalized to the aromatic region.

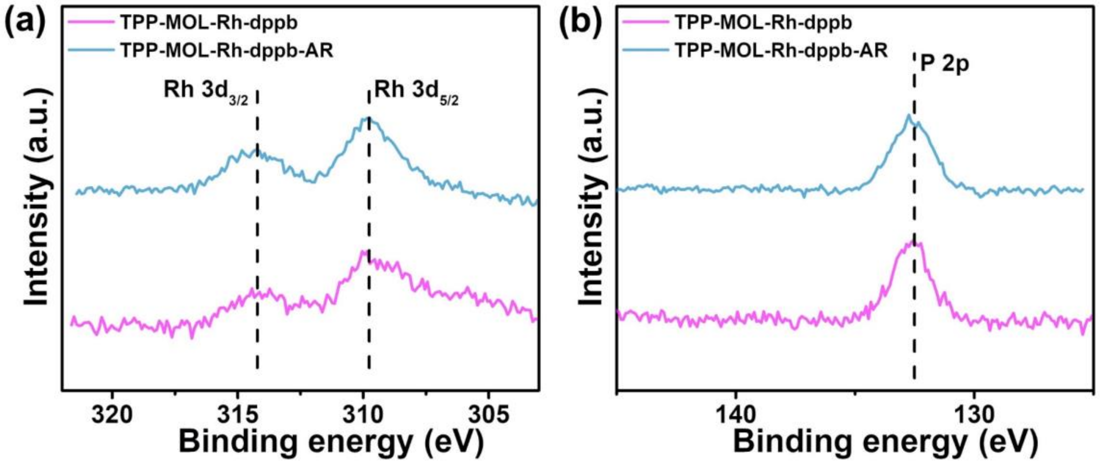

Figure S30 XPS spectra of TPP-MOL-Rh-dppb before and after the reaction.

17. Scope of unsaturated substrates using TPP-MOL-Rh-dppb

We explored the scope of unsaturated substrates using the TPP-MOL-Rh-dppb catalyst under identical PASADENA conditions of 3 bar *p*-H_2 (33%), and the catalyst (0.44 mol% Rh) and substrate (0.182 mmol) amounts were kept consistent.

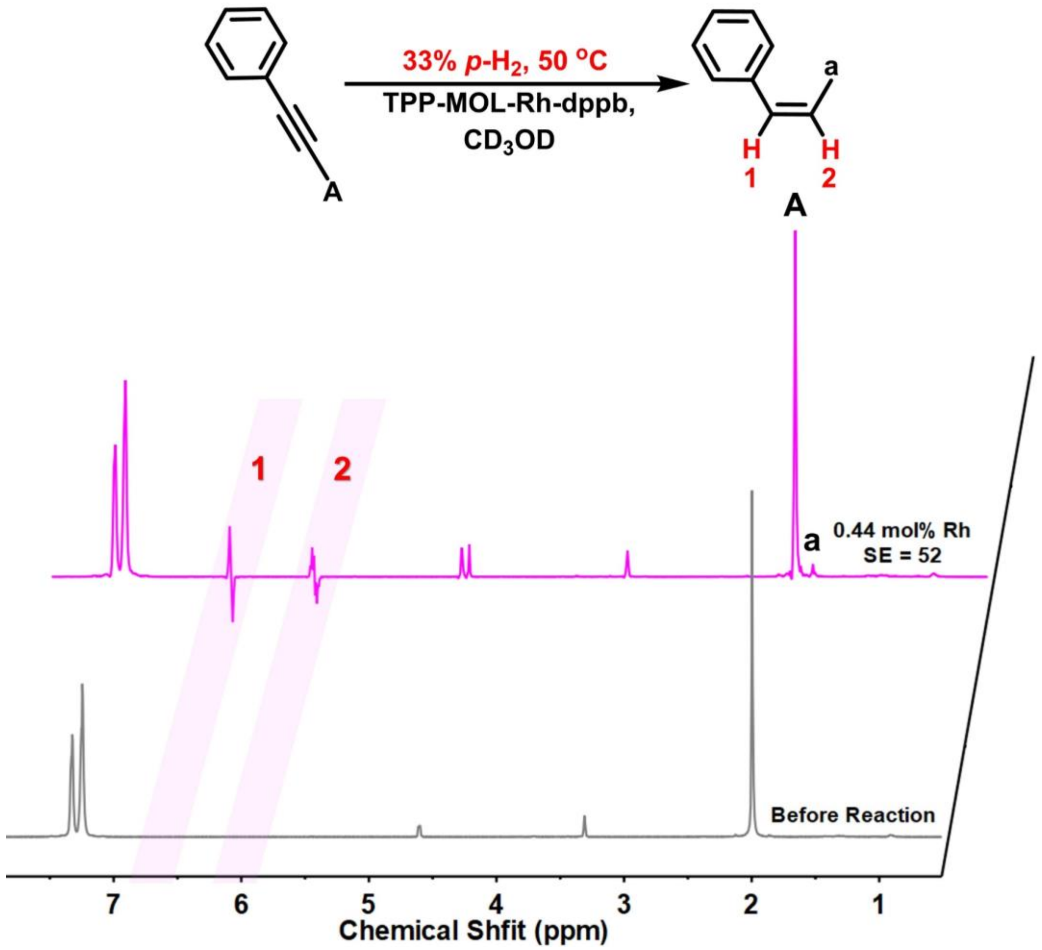

Figure S31 PASADENA ^{1}H-NMR spectra acquired during hydrogenation of 1-phenylpropyne with 33% *p*-H_2 over TPP-MOL-Rh-dppb catalyst (0.44 mol% Rh).

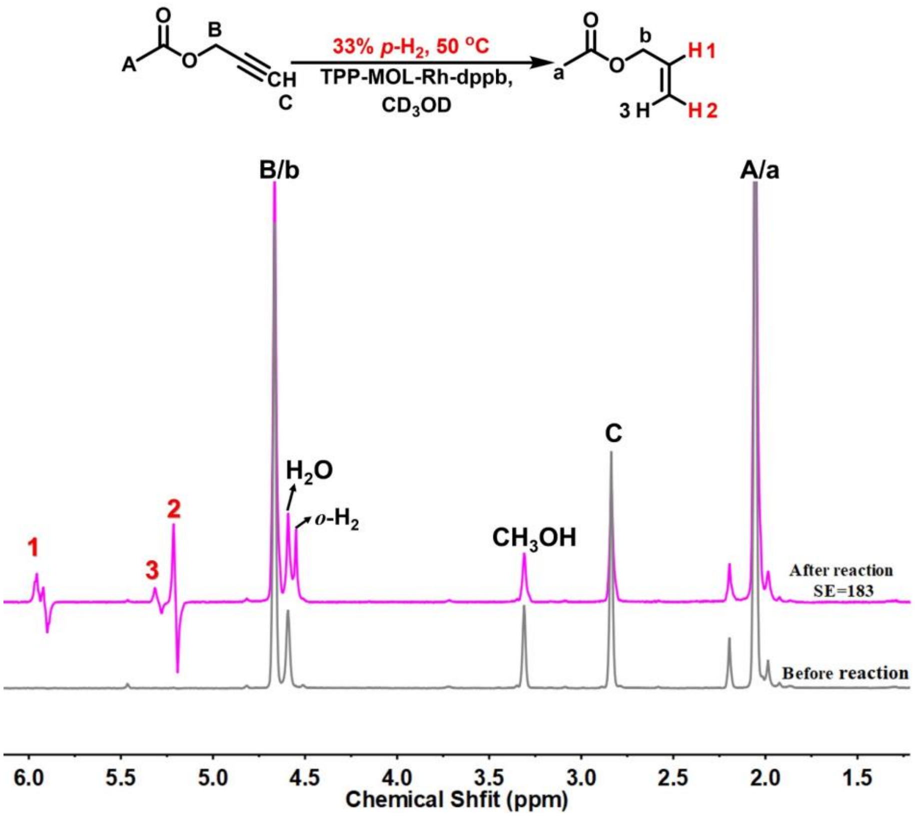

Figure S32 PASADENA ^{1}H-NMR spectra acquired during hydrogenation of propargyl acetate with 33% p-H_2 over TPP-MOL-Rh-dppb catalyst (0.44 mol% Rh). The spectra were normalized using $-CH_3$ peaks.

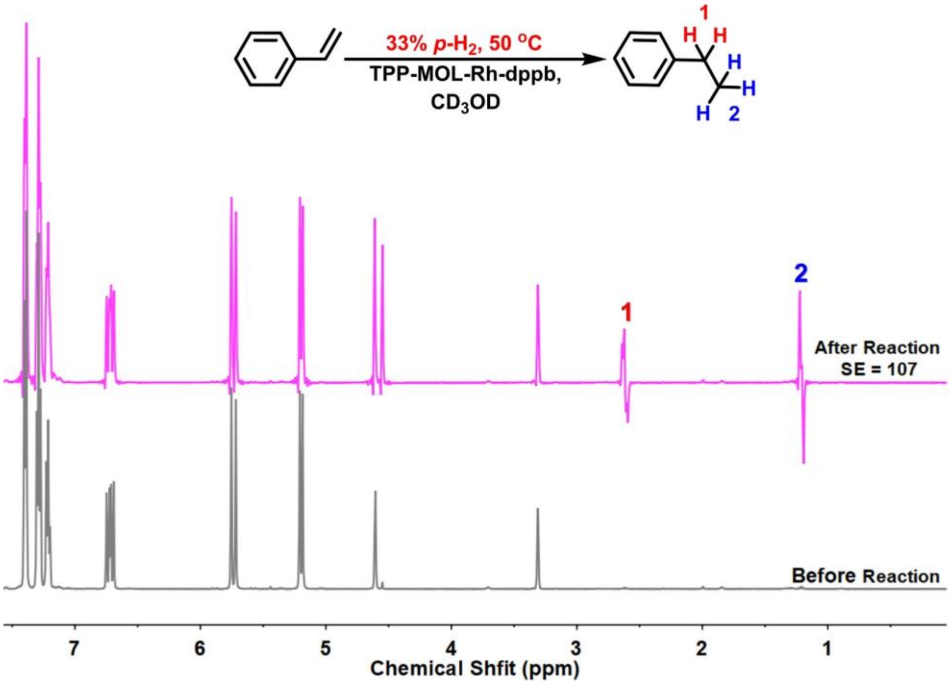

Figure S33 PASADENA ^{1}H-NMR spectra acquired during hydrogenation of styrene with 33% p-H_2 over TPP-MOL-Rh-dppb catalyst (0.44 mol% Rh).

Table S8 HET-PHIP activity for unsaturated substrates by TPP-MOL-Rh-dppb catalyst using 33% p-H_2.

Entry	Substrate (0.182 mmol)	33% p-H_2 [a]	n-H_2 (220 s)[b]	
		Signal Enhancement	Yield of Production (%)	TOF (min^{-1})
1	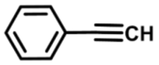	173±15	4.4	2.7
2	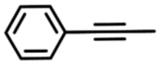	52	7.4	4.6
3	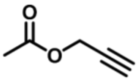	183	1.4	0.9
4	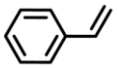	107	1.0	0.6

[a]Reaction conditions: TPP-MOL-Rh-dppb catalyst (0.44 mol% Rh), 0.182 mmol of unsaturated substrates, 0.7 mL of CD_3OD, 50°C, 3 bar p-H_2 (33%) with the flow rate of 28 sccm, other conditions as indicated. [b]The bubbled duration of n-H_2 was 220 s with the flow rate of 28 sccm.

18. Pairwise hydrogenation mechanism

H_2 and D_2 hydrogenation experiments

In a glovebox, the wet TPP-MOL-Rh-dppb catalyst or Rh(COD)(dppb)BF_4 (0.44 mol% Rh), phenylacetylene (20 μL, 0.182 mmol), CH_3OH (0.7 mL) were added into a 25 mL Schlenk tube. The reaction system was purged with H_2 or D_2 gas to a pressure of ~2 bar after vacuum evacuation and were stirred at 50°C for 12 h. The solid was then filtered by centrifugation, and the supernatant was tested by ^{1}H-NMR (CD_3OD) and ^{2}H-NMR (CH_3OH).

19. Simulated PHIP experiment of styrene

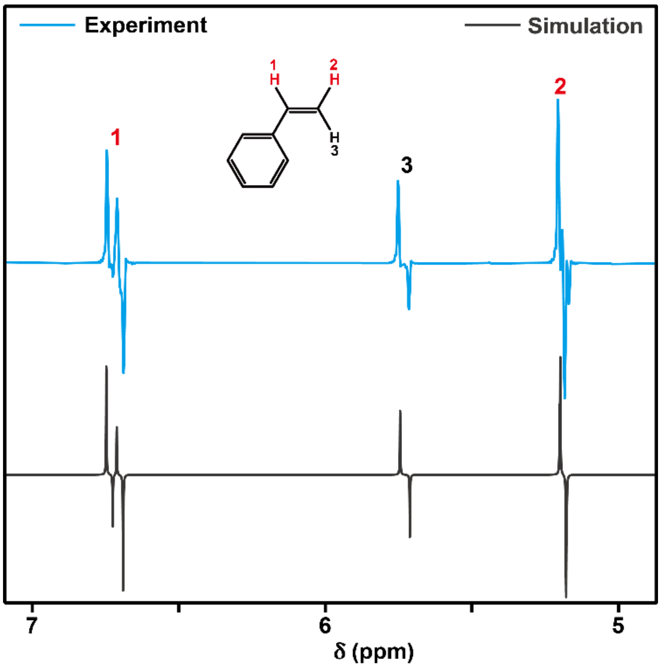

Figure S34 Comparison of experimental and *Spinach* simulated PHIP spectra of styrene. Both spectra only showed results for the protons H1, H2 and H3.

In this section, we further explained the experimental spectrum with the help of density operator theory and *Spinach*[46] simulation calculations.

For convenience, it should be noted that density operators $\boldsymbol{I}_1$, $\boldsymbol{I}_2$ and $\boldsymbol{I}_3$ represent the corresponding protons H1, H2 and H3 of styrene, respectively. The characteristic density operator of parahydrogen is given by:

$$\rho(0) = \frac{1}{4}\boldsymbol{E} - \boldsymbol{I}_1\boldsymbol{I}_2 = \frac{1}{4}\boldsymbol{E} - \boldsymbol{I}_{1z}\boldsymbol{I}_{2z} - \boldsymbol{ZQ}_x \ , \tag{S1}$$

where $\boldsymbol{ZQ}_x$ is the zero-quantum term and can be written as:

$$\boldsymbol{ZQ}_x = \boldsymbol{I}_{1x}\boldsymbol{I}_{2x} + \boldsymbol{I}_{1y}\boldsymbol{I}_{2y} \tag{S2}$$

If the spin system after hydrogenation forms an AA'X three-spin system,[47] the resulting equation for the time-averaged density operator is as follows:

$$\overline{\rho_{AA'X}} = \frac{1}{4}\boldsymbol{E} - \boldsymbol{I}_{1z}\boldsymbol{I}_{2z} - \kappa\{\sin^2\phi\,\boldsymbol{ZQ}_x - \cos\phi\sin\phi(\boldsymbol{I}_{1z} - \boldsymbol{I}_{2z})\boldsymbol{I}_{3z}\} \tag{S3}$$

with

$$\sin^2\phi = \left(\frac{J_{12}}{J'}\right)^2, \ \cos\phi\sin\phi = \left(\frac{J_{12}J_\Delta}{J'^2}\right), \ J_\Delta = \frac{J_{13}-J_{23}}{2}, \ J' = \sqrt{J_{12}{}^2 + J_\Delta{}^2}, \tag{S4}$$

where J_{xy} represents the scalar coupling constants between the protons x and y.

Besides, the κ-value depends on type of the hydrogenation catalyst and crossing polarization effect, and it can be ranged from -1 to 1. In our simulation, κ = -0.4.

Assuming the spin state before acquisition is corresponding to Eq. (S3), the simulated PHIP spectrum can be obtained after applying the 45° hard pulse, as shown in Figure S34. Not only protons H1 and H2, but also proton H3 are hyperpolarized. Under the free evolution of chemical shift and scalar coupling of zero-quantum in AA'X system, effects of converting the initial spin order $\boldsymbol{I}_{1z}\boldsymbol{I}_{2z}$ into the final spin order of $\boldsymbol{I}_{1z}\boldsymbol{I}_{3z}$ and $\boldsymbol{I}_{2z}\boldsymbol{I}_{3z}$ are produced.

20. *Spinach* code for PHIP simulation

```
% PHIP of styrene

% Magnetic field
sys.magnet=11.7;    % 500MHz NMR Spectrometer.

% Spin system
sys.isotopes={'1H','1H','1H'};             % Three-spin system of styrene.

% Chemical shifts
inter.zeeman.scalar={6.72 5.19 5.73};
% Define the chemical shifts of styrene corresponding to protons H1, H2 and H3.

% Scalar couplings
inter.coupling.scalar=cell(3);              % Define the J-coupling network of styrene.

inter.coupling.scalar{1,2}=10.9;
inter.coupling.scalar{1,3}=17.652;
inter.coupling.scalar{2,3}=1.05;

J12=10.9;
J13=17.652;
J23=1.05;
Jd=(J13-J23)/2;
Jp=sqrt(J12^2+Jd^2);

a=(J12/Jp)^2;
c=(J12*Jd)/(Jp*Jp);

% Basis set
```

```
bas.formalism='sphten-liouv';
bas.approximation='none';

% Spinach housekeeping
spin_system=create(sys,inter);
spin_system=basis(spin_system,bas);

% Sequence parameters
parameters.spins={'1H'};

% Define the parameter of catalyst
cata=-0.4;

% % % % % % % % % % % % % % % % %
parameters.rho0=(0.25*state(spin_system,{'E','E','E'},{1,2,3})-state(spin_system,{'Lz'
,'Lz'},{1,2})-…
cata*(a*(state(spin_system,{'L+','L-'},{1,2})+…
state(spin_system,{'L-','L+'},{1,2}))-...
c*(state(spin_system,{'Lz','Lz'},{1,3})-state(spin_system,{'Lz','Lz'},{2,3}))));
% % % % % % % % % % % % % % % % %

parameters.pulse_op=(operator(spin_system,'L+','1H')-...
                     operator(spin_system,'L-','1H'))/(2*1i);
parameters.pulse_angle=pi/4;
parameters.decouple={};
parameters.offset=6.2*500;
parameters.sweep=2000;
parameters.npoints=4096;
parameters.zerofill=8192;
parameters.axis_units='ppm';
parameters.invert_axis=1;

% Simulation
parameters.coil=state(spin_system,'L+','1H');
fid=liquid(spin_system,@hp_acquire,parameters,'nmr');

% Apodization
fid=apodization(fid,'exp-1d',6);

% Fourier transform
spectrum=fftshift(fft(fid,parameters.zerofill));

% Plotting
figure(); plot_1d(spin_system,real(spectrum),parameters);
```

20. References

1. Carroll, T. G.; Hunt, C.; Garwick, R.; Wu, G.; Dobrovetsky, R.; Ménard, G., An untethered C3v-symmetric triarylphosphine oxide locked by intermolecular hydrogen bonding. Chem. Commun. 2019, 55 (26), 3761-3764.
2. Chen, J.; Li, H.; Wang, H.; Song, Y.; Hong, Q.; Chang, K.; Hu, H.; Zhang, S.; Cao, L.; Wang, C., Phosphine-based metal–organic layers to construct single-site heterogeneous catalysts for arene borylation. Chem. Commun. 2023, 59 (54), 8432-8435.
3. Jiang, Y.; Cao, L.; Hu, X.; Ren, Z.; Zhang, C.; Wang, C., Simulating powder X-ray diffraction patterns of two-dimensional materials. Inorg. Chem. 2018, 57 (24), 15123-15132.
4. Cao, L.; Lin, Z.; Peng, F.; Wang, W.; Huang, R.; Wang, C.; Yan, J.; Liang, J.; Zhang, Z.; Zhang, T.; Long, L.; Sun, J.; Lin, W., Self-supporting metal–organic layers as single-site solid catalysts. Angew. Chem. Int. Ed. 2016, 55 (16), 4962-4966.
5. Hövener, J.-B.; Bär, S.; Leupold, J.; Jenne, K.; Leibfritz, D.; Hennig, J.; Duckett, S. B.; von Elverfeldt, D., A continuous-flow, high-throughput, high-pressure parahydrogen converter for hyperpolarization in a clinical setting. NMR Biomed. 2013, 26 (2), 124-131.
6. Pokochueva, E. V.; Kovtunov, K. V.; Salnikov, O. G.; Gemeinhardt, M. E.; Kovtunova, L. M.; Bukhtiyarov, V. I.; Chekmenev, E. Y.; Goodson, B. M.; Koptyug, I. V., Heterogeneous hydrogenation of phenylalkynes with parahydrogen: hyperpolarization, reaction selectivity, and kinetics. Phys. Chem. Chem. Phys. 2019, 21 (48), 26477-26482.
7. Sánchez-Delgado, R. A.; Rosales, M., Kinetic studies as a tool for the elucidation of the mechanisms of metal complex-catalyzed homogeneous hydrogenation reactions. Coord. Chem. Rev. 2000, 196 (1), 249-280.
8. Zhao, E. W.; Maligal-Ganesh, R.; Xiao, C.; Goh, T.-W.; Qi, Z.; Pei, Y.; Hagelin-Weaver, H. E.; Huang, W.; Bowers, C. R., Silica-encapsulated pt-sn intermetallic nanoparticles: a robust catalytic platform for parahydrogen-induced polarization of gases and liquids. Angew. Chem. Int. Ed. 2017, 56 (14), 3925-3929.
9. Song, B.; Choi, D.; Xin, Y.; Bowers, C. R.; Hagelin-Weaver, H., Ultra-low loading Pt/CeO2 catalysts: ceria facet effect affords improved pairwise selectivity for parahydrogen enhanced NMR spectroscopy. Angew. Chem. Int. Ed. 2021, 60 (8), 4038-4042.
10. Burueva, D. B.; Kovtunov, K. V.; Bukhtiyarov, A. V.; Barskiy, D. A.; Prosvirin, I. P.; Mashkovsky, I. S.; Baeva, G. N.; Bukhtiyarov, V. I.; Stakheev, A. Y.; Koptyug, I. V., Selective single-site Pd−In hydrogenation catalyst for production of enhanced magnetic resonance signals using parahydrogen. Chem. Eur. J. 2018, 24 (11), 2547-2553.
11. Wang, W. Y.; Wang, Q.; Chu, Y. Y.; Qi, G. D.; Li, S. H.; Xu, J.; Deng, F., Pairwise stereoselective hydrogenation of propyne on supported Pd-Ag catalysts investigated by parahydrogen-induced polarization. J. Phys. Chem. C 2021, 125 (31), 17144-17154.
12. Salnikov, O. G.; Kovtunov, K. V.; Nikolaou, P.; Kovtunova, L. M.; Bukhtiyarov,

V. I.; Koptyug, I. V.; Chekmenev, E. Y., Heterogeneous parahydrogen pairwise addition to cyclopropane. ChemPhysChem 2018, 19 (20), 2621-2626.
13. Pokochueva, Ekaterina V.; Burueva, D. B.; Kovtunova, L. M.; Bukhtiyarov, A. V.; Gladky, A. Y.; Kovtunov, K. V.; Koptyug, I. V.; Bukhtiyarov, V. I., Mechanistic in situ investigation of heterogeneous hydrogenation over Rh/TiO2 catalysts: selectivity, pairwise route and catalyst nature. Faraday Discuss. 2021, 229 (0), 161-175.
14. Burueva, D. B.; Stakheev, A. Y.; Koptyug, I. V., Pd-based bimetallic catalysts for parahydrogen-induced polarization in heterogeneous hydrogenations. Magn. Reson. 2021, 2 (1), 93-103.
15. Yu, W. W.; Han, H. U.; Jun, X. U.; Feng, D., Hydrogenation reaction on Pd-Cu bimetallic catalysts:a parahydrogen-induced polarization study. Chin. J. Magn. Reson. 2018, 35, 269-279.
16. Han Hu, W. W., Jun Xu, Feng Deng, 1,3-butadienen hydrogenation on supported Pd-Sn bimetallic catalysts investigated by parahydrogen-induced polarization. Chin. J. Magn. Reson. 2022, 39 (2), 133-143.
17. Wang, W. Y.; Hu, H.; Xu, J.; Wang, Q.; Qi, G. D.; Wang, C.; Zhao, X. L.; Zhou, X.; Deng, F., Tuning PdAu bimetallic catalysts for heterogeneous parahydrogen-induced polarization. J. Phys. Chem. C 2018, 122 (2), 1248-1257.
18. Wang, W.; Sun, Q.; Wang, Q.; Li, S.; Xu, J.; Deng, F., Heterogeneous parahydrogen induced polarization on Rh-containing silicalite-1 zeolites: effect of the catalyst structure on signal enhancement. Catal. Sci. Technol. 2022, 12 (14), 4442-4449.
19. Sharma, R.; Bouchard, L.-S., Strongly hyperpolarized gas from parahydrogen by rational design of ligand-capped nanoparticles. Sci. Rep. 2012, 2, 227.
20. Burueva, D. B.; Smirnov, A. A.; Bulavchenko, O. A.; Prosvirin, I. P.; Gerasimov, E. Y.; Yakovlev, V. A.; Kovtunov, K. V.; Koptyug, I. V., Pairwise parahydrogen addition over molybdenum carbide catalysts. Top. Catal. 2020, 63 (1-2), 2-11.
21. Wang, W.; Lewis, R. J.; Lu, B.; Wang, Q.; Hutchings, G. J.; Xu, J.; Deng, F., The role of adsorbed species in 1-butene Isomerization: parahydrogen-induced polarization NMR of Pd-Au catalyzed butadiene hydrogenation. ACS Catal. 2024, 14 (4), 2522-2531.
22. Corma, A.; Salnikov, O. G.; Barskiy, D. A.; Kovtunov, K. V.; Koptyug, I. V., Single-atom gold catalysis in the context of developments in parahydrogen-induced polarization. Chem. Eur. J. 2015, 21 (19), 7012-7015.
23. Zhao, E. W.; Zheng, H.; Ludden, K.; Xin, Y.; Hagelin-Weaver, H. E.; Bowers, C. R., Strong metal-support interactions enhance the pairwise selectivity of parahydrogen addition over Ir/TiO2. ACS Catal. 2016, 6 (2), 974-978.
24. Burueva, D. B.; Bukhtiyarov, A. V.; Prosvirin, I. P.; Baeva, G. N.; Smirnova, N. S.; Mashkovsky, I. S.; Bukhtiyarov, V. I.; Stakheev, A. Y.; Koptyug, I. V., Rh-based intermetallic Rh-In/SiO2 catalyst for parahydrogen-induced polarization. J. Phys. Chem. C 2024, 128 (15), 6319-6327.
25. Skovpin, I. V.; Kovtunova, L. M.; Nartova, A. V.; Kvon, R. I.; Bukhtiyarov, V. I.; Koptyug, I. V., Anchored complexes of rhodium and iridium for the hydrogenation of alkynes and olefins with parahydrogen. Catal. Sci. Technol. 2022, 12 (10), 3247-3253.

26. Zhivonitko, V. V.; Skovpin, I. V.; Szeto, K. C.; Taoufik, M.; Koptyug, I. V., Parahydrogen-Induced polarization study of the silica-supported vanadium oxo organometallic catalyst. J. Phys. Chem. C 2018, 122 (9), 4891-4900.
27. Skovpin, I. V.; Zhivonitko, V. V.; Prosvirin, I. P.; Khabibulin, D. F.; Koptyug, I. V., Gas-phase hydrogenation with parahydrogen over immobilized Vaska's complex. Z. Phys. Chem. 2017, 231 (3), 575-592.
28. Nartova, A. V.; Kvon, R. I.; Kovtunova, L. M.; Skovpin, I. V.; Koptyug, I. V.; Bukhtiyarov, V. I., XPS and HRTEM elucidation of the diversity of titania-supported single-site Ir catalyst performance in spin-selective propene hydrogenation. Int. J. Mol. Sci. 2023, 24 (21), 15643-15656.
29. Skovpin, I. V.; Zhivonitko, V. V.; Kaptein, R.; Koptyug, I. V., Generating parahydrogen-induced polarization using immobilized iridium complexes in the gas-phase hydrogenation of carbon-carbon double and triple bonds. Appl. Magn. Reson. 2013, 44 (1-2), 289-300.
30. Skovpin, I. V.; Zhivonitko, V. V.; Koptyug, I. V., Parahydrogen-induced polarization in heterogeneous hydrogenations over silica-immobilized Rh complexes. Appl. Magn. Reson. 2011, 41 (2-4), 393-410.
31. Kovtunov, K. V.; Zhivonitko, V. V.; Corma, A.; Koptyug, I. V., Parahydrogen-induced polarization in heterogeneous hydrogenations catalyzed by an immobilized Au(III) complex. J. Phys. Chem. Lett. 2010, 1 (11), 1705-1708.
32. Wang, W.; Xu, J.; Zhao, Y.; Qi, G.; Wang, Q.; Wang, C.; Li, J.; Deng, F., Facet dependent pairwise addition of hydrogen over Pd nanocrystal catalysts revealed via NMR using para-hydrogen-induced polarization. Phys. Chem. Chem. Phys. 2017, 19 (14), 9349-9353.
33. Salnikov, O. G.; Chukanov, N. V.; Kovtunova, L. M.; Bukhtiyarov, V. I.; Kovtunov, K. V.; Shchepin, R. V.; Koptyug, I. V.; Chekmenev, E. Y., Heterogeneous 1H and 13C parahydrogen-induced polarization of acetate and pyruvate esters. ChemPhysChem 2021, 22 (13), 1389-1396.
34. Zhao, T. Y.; Lapak, M. P.; Behera, R.; Zhao, H.; Ferrer, M.-J.; Weaver, H. E. H.; Huang, W.; Bowers, C. R., Perpetual hyperpolarization of allyl acetate from parahydrogen and continuous flow heterogeneous hydrogenation with recycling of unreacted propargyl acetate. J. Magn. Reson. Open 2022, 12-13, 100076.
35. Hale, W. G.; Zhao, T. Y.; Choi, D.; Ferrer, M.-J.; Song, B.; Zhao, H.; Hagelin-Weaver, H. E.; Bowers, C. R., Toward continuous-flow hyperpolarisation of metabolites via heterogenous catalysis, side-arm-hydrogenation, and membrane dissolution of parahydrogen. ChemPhysChem 2021, 22 (9), 822-827.
36. Salnikov, O. G.; Burueva, D. B.; Kovtunova, L. M.; Bukhtiyarov, V. I.; Kovtunov, K. V.; Koptyug, I. V., Mechanisms of methylenecyclobutane hydrogenation over supported metal catalysts studied by parahydrogen-induced polarization technique. ChemPhysChem 2022, 23 (7), e202200072.
37. Salnikov, O. G.; Kovtunov, K. V.; Koptyug, I. V., Production of catalyst-free hyperpolarised ethanol aqueous solution via heterogeneous hydrogenation with parahydrogen. Sci. Rep. 2015, 5, 13930.
38. Irfan, M.; Eshuis, N.; Spannring, P.; Tessari, M.; Feiters, M. C.; Rutjes, F. P. J. T.,

Liquid-phase parahydrogen-induced polarization (PHIP) with ligand-capped platinum nanoparticles. J. Phys. Chem. C 2014, 118 (24), 13313-13319.
39. Glöggler, S.; Grunfeld, A. M.; Ertas, Y. N.; McCormick, J.; Wagner, S.; Schleker, P. P. M.; Bouchard, L.-S., A nanoparticle catalyst for heterogeneous phase para-hydrogen-induced polarization in water. Angew. Chem. Int. Ed. 2015, 54 (8), 2452-2456.
40. Alshehri, A.; Tickner, B. J.; Iali, W.; Duckett, S. B., Enhancing the NMR signals of plant oil components using hyperpolarisation relayed via proton exchange. Chem. Sci. 2023, 14 (36), 9843-9853.
41. Iqbal, N.; Brittin, D. O.; Daluwathumullagamage, P. J.; Alam, M. S.; Senanayake, I. M.; Gafar, A. T.; Siraj, Z.; Petrilla, A.; Pugh, M.; Tonazzi, B.; Ragunathan, S.; Poorman, M. E.; Sacolick, L.; Theis, T.; Rosen, M. S.; Chekmenev, E. Y.; Goodson, B. M., Toward next-generation molecular imaging with a clinical low-field (0.064 T) point-of-care MRI scanner. Anal. Chem. 2024, 96 (25), 10348-10355.
42. Ellermann, F.; Saul, P.; Hoevener, J.-B.; Pravdivtsev, A. N., Modern manufacturing enables magnetic field cycling experiments and parahydrogen-induced hyperpolarization with a benchtop NMR. Anal. Chem. 2023, 95 (15), 6244-6252.
43. Alam, M. S.; Li, X.; Brittin, D. O.; Islam, S.; Deria, P.; Chekmenev, E. Y.; Goodson, B. M., Anomalously large antiphase signals from hyperpolarized orthohydrogen using a MOF-based SABRE catalyst. Angew. Chem. Int. Ed. 2023, 62 (8), e202213581.
44. Tickner, B. J.; Dennington, M.; Collins, B. G.; Gater, C. A.; Tanner, T. F. N.; Whitwood, A. C.; Rayner, P. J.; Watts, D. P.; Duckett, S. B., Metal-mediated catalytic polarization transfer from para-hydrogen to 3,5-dihalogenated pyridines. ACS Catal. 2024, 14 (2), 994-1004.
45. Rayner, P. J.; Burns, M. J.; Olaru, A. M.; Norcott, P.; Fekete, M.; Green, G. G. R.; Highton, L. A. R.; Mewis, R. E.; Duckett, S. B., Delivering strong 1H nuclear hyperpolarization levels and long magnetic lifetimes through signal amplification by reversible exchange. Proc. Natl. Acad. Sci. USA 2017, 114 (16), E3188-E3194.
46. Hogben, H. J.; Krzystyniak, M.; Charnock, G. T.; Hore, P. J.; Kuprov, I., Spinach–a software library for simulation of spin dynamics in large spin systems. J. Magn. Reson. 2011, 208 (2), 179-194.
47. Green, R. A.; Adams, R. W.; Duckett, S. B.; Mewis, R. E.; Williamson, D. C.; Green, G. G., The theory and practice of hyperpolarization in magnetic resonance using parahydrogen. Prog. Nucl. Magn. Reson. Spectrosc. 2012, 67, 1-48.
.
